# The miR9863 Family Regulates Distinct *Mla* Alleles in Barley to Attenuate NLR Receptor-Triggered Disease Resistance and Cell-Death Signaling

**DOI:** 10.1371/journal.pgen.1004755

**Published:** 2014-12-11

**Authors:** Jie Liu, Xiliu Cheng, Da Liu, Weihui Xu, Roger Wise, Qian-Hua Shen

**Affiliations:** 1 State Key Laboratory of Plant Cell and Chromosome Engineering, Centre for Molecular Agrobiology, Institute of Genetics and Developmental Biology, Chinese Academy of Sciences, Beijing, China; 2 University of Chinese Academy of Sciences, Beijing, China; 3 Department of Plant Pathology & Microbiology, Center for Plant Responses to Environmental Stresses, Iowa State University, Ames, Iowa, United States of America; 4 Corn Insects and Crop Genetics Research, USDA-Agricultural Research Service, Iowa State University, Ames, Iowa, United States of America; Virginia Tech, United States of America

## Abstract

Barley (*Hordeum vulgare* L.) *Mla* alleles encode coiled-coil (CC), nucleotide binding, leucine-rich repeat (NB-LRR) receptors that trigger isolate-specific immune responses against the powdery mildew fungus, *Blumeria graminis* f. sp. *hordei* (*Bgh*). How *Mla* or NB-LRR genes in grass species are regulated at post-transcriptional level is not clear. The microRNA family, miR9863, comprises four members that differentially regulate distinct *Mla* alleles in barley. We show that miR9863 members guide the cleavage of *Mla1* transcripts in barley, and block or reduce the accumulation of MLA1 protein in the heterologous *Nicotiana benthamiana* expression system. Regulation specificity is determined by variation in a unique single-nucleotide-polymorphism (SNP) in mature miR9863 family members and two SNPs in the *Mla* miR9863-binding site that separates these alleles into three groups. Further, we demonstrate that 22-nt miR9863s trigger the biogenesis of 21-nt phased siRNAs (phasiRNAs) and together these sRNAs form a feed-forward regulation network for repressing the expression of group I *Mla* alleles. Overexpression of miR9863 members specifically attenuates MLA1, but not MLA10-triggered disease resistance and cell-death signaling. We propose a key role of the miR9863 family in dampening immune response signaling triggered by a group of MLA immune receptors in barley.

## Author Summary

Plants rely on cell-surface and intracellular immune receptors to sense pathogen invasion and to mediate defense responses. However, uncontrolled activation of immune responses is harmful to plant growth and development. Small RNAs have recently been shown to fine-tune the expression of intracellular immune receptors and contribute to the regulation of defense signaling in dicot plants, while similar processes have not been well documented in monocot grain crops, such as barley and wheat. Here, we show that, in barley, some members of the miR9863 family target a subset of *Mla* alleles that confer race-specific disease resistance to the powdery mildew fungus. These miRNAs act on *Mla* transcripts by cleavage and translational repression. Production of a type of trans-acting small RNAs, designated as phasiRNAs, enhances the effects of miRNA regulation on *Mla* targets. We propose that *Mla*-mediated immune signaling is fine-tuned by the miRNAs at later stage of MLA activation to avoid overloading of immune responses in barley cells.

## Introduction

Plants have evolved two major classes of immune receptors for pathogen recognition and defense activation [1]. Pattern recognition receptors (PRRs) detect conserved pathogen associated molecular patterns (PAMP) and trigger PAMP-triggered immunity (PTI) [2], whilst intracellular NB-LRR (NLR) receptors recognize isolate-specific pathogen effectors and trigger effector-triggered immunity (ETI), often accompanied by a hypersensitive reaction (HR) [3], [4]. PTI and ETI are believed to share highly overlapping signaling networks for effective immune responses [5]. A plant genome may contain hundreds of genes encoding NLR receptors [6]. Improper activation of some NLR receptors is accompanied by autoimmunity that is harmful to plant growth [7], [8], thus, expression of *NLR* genes is likely under tight control at different levels.

Plant small RNAs are noncoding RNAs that largely fall into two groups, microRNAs (miRNAs) and small interfering RNAs (siRNAs). Plant small RNAs have important roles in plant growth and development, abiotic stress responses and antiviral defense responses [9]–[11]. Increasing evidence indicates that plant small RNAs also participate in PTI and ETI responses against bacterial and fungal pathogens [12], [13]. For example, the miR393 and miR393* pair are involved in Arabidopsis PTI responses against *Pseudomonas syringae* pv. tomato (*Pst*) [14]–[16] and hvu-miR398 is regulated by the barley *Mla* NLR immune receptor, influencing ETI responses to the powdery mildew fungus [17]. Further, the natural antisense (NAT) RNA, nat-siRNAATGB2, and a long siRNA, lsiRNA-1, contribute to Arabidopsis RPS2-mediated ETI responses to *Pst* (*avrRpt2*) [18], [19]. Interestingly, recent examples indicate that plant small RNA pathways are required for defense responses against *Verticllium dahliae* [20] and are themselves targets of effectors secreted by oomycetes and fungi [21], [22].

MicroRNAs are 21–24 nucleotides (nt) endogenous small RNAs that repress gene expression in plants and animals [23]. In plants, the majority of miRNAs originating from *MIR* genes are processed by the DICER-LIKE1 (DCL1) enzyme from long primary miRNA transcripts that form imperfect stem-loop structures [9], [10], [24]. The mature miRNAs are loaded into an ARGONAUTE (AGO) protein to form functional RNA-induced silencing complex (RISC) for target gene silencing [25]. Plant miRNAs mediate target silencing through at least two modes of action, i.e., target mRNA cleavage and translational repression [24], [26]. Unlike animal miRNAs, plant miRNAs are highly complementary to their targets and earlier studies have shown that plant miRNAs guide target mRNA cleavage via AGO slicers [27]–[29], thus guiding RNA cleavage was believed to be the major activity of plant miRNAs [9]. Increasing evidence has indicated that plant miRNAs also inhibit target mRNA translation [30]–[36], and in some cases, plant miRNAs may regulate targets through both mRNA cleavage and translation inhibition [30], [32], [35]. Recent work has demonstrated that the *ALTERED MERISTEM PROGRAM1* (*AMP1*)-dependent activity of miR398 in repressing translation of the *CSD2* gene on the ER [37], further clarifying the role of plant miRNAs in inhibition of target gene translation.

The role of plant miRNAs in modulating immunity has recently been shown in diverse plant species, particularly their involvement in post-transcriptional control of NLR receptors [38]–[41]. In *Medicago truncatula*, highly abundant miRNA families, e.g., miR2109, miR2118 and miR1507, act as master regulators and target sites encoding highly conserved domains of a large group of NLR receptors [38], and this regulatory circuit can be extended into non-legume plant species, e.g., potato [38]. In *N. benthamiana*, two miRNAs, miR6019 and miR6020, were shown to regulate the TIR-NB-LRR (TNL)-type N receptor gene and to attenuate N-mediated resistance against *Tobacco mosaic virus* [39], while in other *Solanaceae* species the miR482/2118 superfamily and additional miRNA families target both the TNL- and CC-NB-LRR (CNL)-type receptor genes [39], [40]. Moreover, ath-miR472, a miR482-related Arabidopsis miRNA, was recently reported to act together with *RDR6* to target *CNL* genes, including *RPS5* and *SUMM2*, and to modulate both PTI and ETI against bacterial pathogens through post-transcriptional regulation of a subset of *CNL* genes [41].

Plant *NLR*-targeting miRNAs are in general of 22-nt in length, and they are able to trigger the production of secondary siRNAs that form a feed-forward regulation loop to amplify the suppression effect on *NLR* genes [38]–[42]. These *trans*- or *cis*-acting small interfering RNAs are usually in phase with the 5′-end of the cleavage site of the target transcripts, thus called phased secondary siRNAs (phasiRNAs) [43]. While miRNAs of 22-nt rather than 21-nt are believed to be the preferential triggers of phasiRNAs [44]–[46], the asymmetrical miRNA duplex structure is also important in triggering secondary siRNAs [47]. Despite the involvement of miRNAs and phasiRNAs in modulating plant immunity against pathogens in diverse plant species, the functional relevance of *NLR*-targeting miRNAs and the secondary siRNAs has not been well addressed in plant-fungal interactions, particularly for *Poaceae* species.

In barley, *Mildew resistance locus a* (*Mla*) contains a large number of alleles encoding NLR immune receptors that have verified or anticipated race-specific disease resistance activity against the powdery mildew fungus, *Blumeria graminis* f. sp. *hordei* (*Bgh*) [48]. Recent findings indicate that upon pathogen recognition MLAs dynamically integrate PTI and ETI immune signaling pathways in both nuclear and cytoplasmic compartments to mediate defense responses [49], [50]. Upon activation MLA associates in the nucleus with transcription factors (TFs), i.e., WRKY and MYB TFs, to initiate extensive transcriptional reprogramming that is critical for effective immune responses [51]–[55], whereas MLA triggers cell-death signaling in the cytosol through as yet unknown components that are probably conserved in monocots and dicots [56]–[58]. Recent data show that *Mla* mediates control of barley miR398 that represses chloroplast *Hv*SOD1 accumulation, thus de-repressing HR cell-death signaling in response to *Bgh* challenge [17]. Previous studies have also shown that the activity of some MLAs is tightly regulated at post-translational level by the co-chaperone *Required for Mla12 resistance 1* (*RAR1*) [59]–[61]. Nevertheless, whether and how *Mla* alleles are regulated at post-transcriptional level in barley remains unknown.

Here, we show that members of the *Triticeae*-specific miR9863 family differentially regulate a subset of barley *Mla* alleles at the post-transcriptional level. Unique single-nucleotide-polymorphism (SNP) sites in mature miR9863s and the miR9863-binding site of *Mla* alleles determine regulation specificity. Furthermore, we demonstrate in barley and *N. benthamiana* that miR9863s trigger the biogenesis of phasiRNAs and together these small RNAs form a regulation network for controlling *Mla* expression. Overexpression of miR9863 members specifically attenuates MLA1, but not MLA10-mediated disease resistance against *Bgh* in barley or induction of cell-death signaling in *N. benthamiana*, signifying the miR9863 family in fine-tuning immunity triggered by MLA receptors.

## Results

### Identification of the miR9863 family in barley and wheat

**Figure 1:**
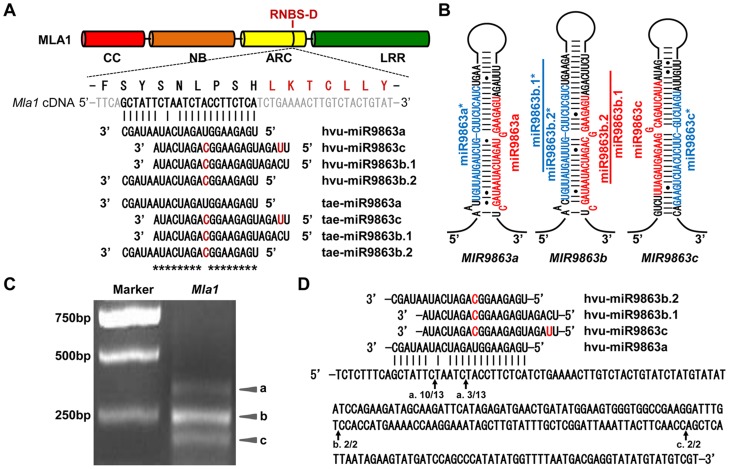
miR9863 family members target *Mla1* transcripts in barley. (**A**) Alignment of *Mla1* transcript with miR9863 family members from barley and wheat. Shown above the alignment are the MLA1 domain structure (CC, NB, ARC and LRR), the amino acid sequence of the miRNA target region and the neighboring RNBS-D motif (in red). hvu, *Hordeum vulgare*; tae, *Triticum aestivum*. (**B**) Predicted secondary structures of *hvu-MIR9863a*, *hvu -MIR9863b* and *tae-MIR9863c* precursors. Since *hvu-MIR9863c* could not be amplified from barley cultivars (i.e. Pallas and Golden promise), we used *tae-MIR9863c* for generating miR9863c in this study. The *hvu-MIR9863b* precursor generates both miR9863b.1 and miR9863b.2 that partially overlap with each other. (**C**) Ethidium bromide stained agarose gel of 5′ RACE products obtained from the barley line P01 (a Pallas near-isogenic line containing the *Mla1* allele). 5′ RACE products were generated by using total RNAs isolated from P01, and ‘a’, ‘b’, and ‘c’ indicate three different products derived from different cleavage sites (see below). (**D**) Cleavage sites in the *Mla1* transcript confirmed by the sequencing of 5′ RACE products. Arrows indicate the cleavage sites, ‘a’, ‘b’ and ‘c’, corresponding to three products shown in (**C**); numbers below the arrows show frequency of clones with matching 5′-RACE product from this site out of total clones confirmed by sequencing.

To investigate the role of miRNAs in barley (*Hordeum vulgare* L.) resistance to the powdery mildew pathogen, *Bgh*, we constructed small RNA (sRNA) libraries using samples derived from healthy and *Bgh*-infected leaves of the near-isogenic line P01 that harbors the *Mla1* resistance allele [62]. Deep sequencing and data analyses of the sRNA libraries identified miRNAs that were either up- or down-regulated after *Bgh* infection. We searched in our sequence data for miRNAs potentially targeting *Mla* alleles and found that members of one miRNA family are complementary to the coding sequence for the region adjacent and with two-amino acids overlapping with the RNBS-D motif in *Mla1* (Fig. 1A and 1D). Amino acid sequence alignment indicated that this potential miRNA target site is highly conserved among *Mla* alleles, as well as some *R* genes from wheat and related species (S1 Figure). The same miRNA family was also ascertained by an independent small RNA sequencing project [63] and described in previous small RNA deep sequencing studies in barley and bread wheat (*Triticum aestivum* L.). This family was originally designated in wheat as miR2009 with four members, i.e., miR2009a, miR2009b, miR2009c and miR2009d (S1 Table) [64]–[66]. However, the plant miR2009 was not formally included in miRBase20.0, while a sea urchin miRNA was already registered as miR-2009 [67]. Thus, the name of the wheat miR2009 was most likely assigned arbitrarily and thus bears the same name as that of the sea urchin miRNA. Therefore, we have renamed the plant miRNA family with its own unique designator in miRBase20.0, miR9863. We set out to search for barley and wheat EST sequences containing miR9863 members, and to BLAST wheat and barley genome databases. We found that miR9863a could be aligned to the wheat EST, CK193889, while miR9863c aligned to another wheat EST, DR736484 (S2A Figure). Interestingly, miR9863b and miR9863d overlap by 17 nucleotides, and cluster with miR9863a on the same wheat EST (i.e., CK193889) with 244 nt in-between (S2A Figure; S1 Table). In barley, miR9863b and miR9863d also align to one barley cDNA sequence, AK364228, overlapping by 17 nt (S2A Figure; S1 Table). Since miR9863b and miR9863d are two miRNAs that likely resulted from overlapping processing of the same locus, we designated these two miRNAs as miR9863b.1 and miR9863b.2 (Fig. 1A; S1 Table) [68]. The miRNA and miRNA* sequence and the flanking EST sequences together can form a typical hairpin structures, with the miRNA and miRNA* on the stem region (Fig. 1B; S2A Figure). Thus, these analyses indicate that the miR9863 family is present in the *Triticeae*, and members of this family could be potential regulators of *Mla* alleles.

Next we attempted to isolate the precursors of the miR9863 family from both barley and wheat. While we could readily amplify all three progenitors from wheat, i.e., *MIR9863a*, *MIR9863c* and *MIR9863b*, we could only amplify two progenitors from barley, *MIR9863a* and *MIR9863b*, but not *MIR9863c* (Fig. 1B). *MIR9863c* also was not present in an independent deep sequencing data set of barley cv. Morex, both from non-inoculated and *Bgh* inoculated leaves [63]. It is possible that miR9863c may be genotype specific in barley, or an intron-derived microRNA.

Sequence analysis revealed that the *MIR9863a* progenitor from barley and wheat are highly conserved, and the same is true for *MIR9863b* (S2A Figure). Since *MIR9863a* and *MIR9863b* are aligned in tandem on the same wheat EST (CK193889), this suggests the existence of a wheat polycistron of *MIR9863a*-*MIR9863b*, which may not be present in barley (S2A Figure). To test whether these potential miRNA progenitors can generate miRNAs, we transiently expressed them in *N. benthamiana* by *Agrobacterium*-mediated infiltration and found the accumulation of 22-nt mature miRNAs in all cases (S2B Figure). Further, we examined the expression of the miR9863 family in different plant species (S3 Figure). Interestingly, we found that they are expressed in all tested barley and bread wheat cultivars, but do not accumulate in other tested monocots, e.g., *Brachypodium distachyon*, rice (*Oryza sativa*) and maize (*Zea mays*), nor in the dicots *N. benthamiana* and *A. thaliana* (S3 Figure). Although searching of the monocot small RNA library using miR9863 sequences found some sorghum small RNAs matching miR9863a [63], however, we could not further retrieve any flanking sequences from sorghum EST or genome database that can form a miRNA precursor structure [69]. Furthermore, searching of several sRNA libraries from *Brachypodium distachyon* [66], [70], [71], which has been served as a model for the *Triticeae* tribe, did not find any matching sequences to the miR9863 sequences. We also screened NCBI genome and EST databases of rice and Arabidopsis using the mature miR9863 sequences (allowing no more than 2 mismatches), but failed to identify a single match. Together, these suggest that the *MIR9863* loci may be *Triticeae*-specific innovation.

### The miR9863 family targets barley *Mla1* for cleavage

To examine the function of the miR9863 members in directing *Mla1* transcript cleavage in barley, we conducted 5′-RACE using *Bgh* infected P01 (harboring the *Mla1* allele) leaf samples. We could detect three major cleavage products from *Mla1* transcripts after gel-electrophoresis (Fig. 1C, marked by ‘a’, ‘b’, and ‘c’). Sequencing of clones derived from band ‘a’ revealed two cleavage positions within the miR9863-binding site in *Mla1* transcripts (Fig. 1C and 1D). To test the specificity of miR9863a binding, we conducted site-directed mutagenesis by replacing the ‘T’ with an ‘A’ at the position next to the 1^st^ cleavage site, and ‘CT’ with ‘GA’ across the 2^nd^ cleavage site, resulting in *Mla1*-T1266A and *Mla1*-CT1270GA. This should result in a loop at the respective cleavage site in the miRNA/target duplex (S4A Figure). We next determined the efficiency of miR9863a on wild-type (WT) *Mla1* and the two *Mla1* variants by transient expression in *N. benthamiana*. Upon co-expression of *MIR9863a* with WT *Mla1*, MLA1 protein was decreased to a level that was barely detectable by Western blot analysis (S4B Figure, lane 2). By contrast, MLA1-CT1270GA or MLA1-T1266A protein abundance was not affected or only slightly reduced (S4B Figure, lanes 3 and 4, 5 and 6, respectively), compared to the empty vector (EV) controls (S4B Figure, lane 1). These results suggest the strict pairing at the 10^th^ to 11^th^ nt and the 15^th^ nt positions in the miR9863a-binding site of *Mla1* is necessary for proper regulation of miR9863a. Sequencing of clones derived from band ‘b’ and ‘c’ revealed two additional cleavage sites downstream of the miR9863-binding position (Fig. 1C and 1D). We speculate that these two cleavage sites were derived from in-phase degradation triggered by secondary siRNAs (see below). In summary, the potentially *Triticeae*-specific miR9863 family has four members, and some members of this miRNA family direct the cleavage of *Mla1* transcripts at the miRNA-binding site.

### miR9863 family members differentially regulate *Mla1*

**Figure 2:**
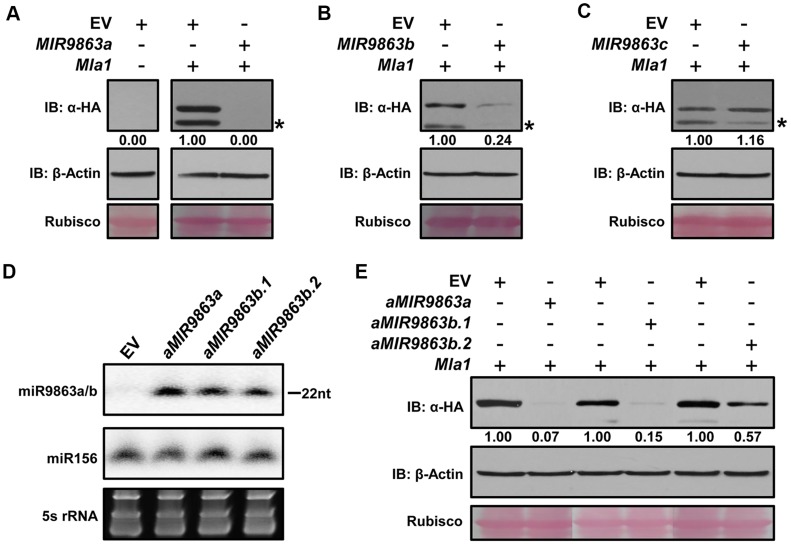
miR9863a, miR9863b.1 and miR9863b.2, but not miR9863c, regulate *Mla1*. (**A**) to (**C**) miR9863 family members differentially regulate *Mla1* gene shown by transient gene expression assay in *N. benthamiana*. Agrobacteria strain expressing *Mla1*-3HA were co-infiltrated into *N. benthamiana* leaves with strains containing the respective miRNA precursors, *hvu-MIR9863a* (**A**), *hvu-MIR9863b* (**B**) or *tae-MIR9863c* (**C**). Western blotting was used to detect MLA1-3HA or actin levels at 36 hours post Agro-infiltration (hpai); and the number below the blots indicates relative protein levels as calculated by Image J. Empty vector (EV) was used as a negative control and rubisco served as a loading control. The asterisks indicate non-specific signals. Experiments were performed at least two times with similar results. (**D**) Expression of mature miR9863a, miR9863b.1 and miR9863b.2 using artificial *MIRNA* backbone. The mature miR9863a, miR9863b.1 or miR9863b.2 sequences were engineered into the Arabidopsis *MIR173* precursor (see S5 Figure) and then artificial *MIRNA* precusors (*aMIRNA*) were expressed in *N. benthamiana* by Agro-infiltration. The levels of mature miR9863s were detected by RNA gel blot analysis at 36 hpai. miR156 and 5S rRNA were employed as loading controls. (**E**) Determination of the regulation efficiency of miR9863a, miR9863b.1 or miR9863b.2 on *Mla1*. The *aMIR9863a*, *aMIR9863b.1* or *aMIR9863b.2* was respectively co-expressed with *Mla1* in *N. benthamiana*, and MLA1 protein levels were determined at 36 hpai by immunoblotting.

Functional differentiation is usually observed among different miRNA members that belong to the same family [31], [72], [73]. To determine the specificity of individual miR9863 members for *Mla1*, we co-expressed C-terminal HA-tagged MLA1 with progenitor *MIR9863a*, *MIR9863b* or *MIR9863c*, respectively, in *N. benthamiana* through *Agrobacterium*-mediated infiltration (Fig. 1B; Fig. 2). MLA1 accumulation was fully or significantly blocked by the expression of *MIR9863a* or *MIR9863b* but not by *MIR9863c*, compared to EV control (Fig. 2A, 2B and 2C). Since *MIR9863b* should simultaneously generate miR9863b.1 and miR9863b.2 (Fig. 1B), we could not differentiate the effect of these two miRNAs (Fig. 2B). To address this, we utilized the Arabidopsis precursor *Ath-MIR173* as a backbone and constructed artificial miRNA expression vectors, *aMIR9863a*, *aMIR9863b.1* and *aMIR9863b.2* (S5 Figure) [17], [44], [74]. In *N. benthamiana* these *aMIRNA* precursors could respectively generate miR9863a, miR9863b.1, or miR9863b.2 to a comparable level (Fig. 2D). Further co-expression of *aMIR9863a* with *Mla1* fully repressed MLA1 accumulation in *N. benthamiana* (Fig. 2E, lanes 1 and 2). Importantly, *aMIR9863b.1* expression could largely block MLA1 accumulation whilst *aMIR9863b.2* expression has only a subtle effect, resulting in accumulation of MLA1 at ∼15%, or ∼60% of that in EV control, respectively (Fig. 2E, lanes 3 and 4, lanes 5 and 6). These results demonstrate that *Mla1* is differentially regulated by miR9863 members, with the highest efficiency specified by miR9863a and miR9863b.1, less so by miR9863b.2, and none by miR9863c.

It is interesting to note that miR9863a and miR9863b.2 differ markedly in their regulation of *Mla1*, but have only a single nucleotide difference (Fig. 1A; S6A Figure). To further characterize this observation, we introduced a ‘T’ to ‘C’ point mutation in the *MIR9863a* progenitor, resulting in *MIR9863a*-T9C that should produce a miR9863a variant sequentially same as miR9863b.2 (S6B Figure). Indeed, unlike *MIR9863a* that fully blocked MLA1 accumulation, *MIR9863a-T9C* co-expression with *Mla1* led to high MLA1 level to ∼80% of that in EV control (S6C Figure, panel a); RNA gel-blotting detected equal expression level of the respective miRNAs (S6C Figure, panel b). These data indicate that a single nucleotide difference between miR9863a and miR9863b.2 can influence the regulation of *Mla1*.

### miR9863 family members regulate only a subset of *Mla* genes

**Figure 3:**
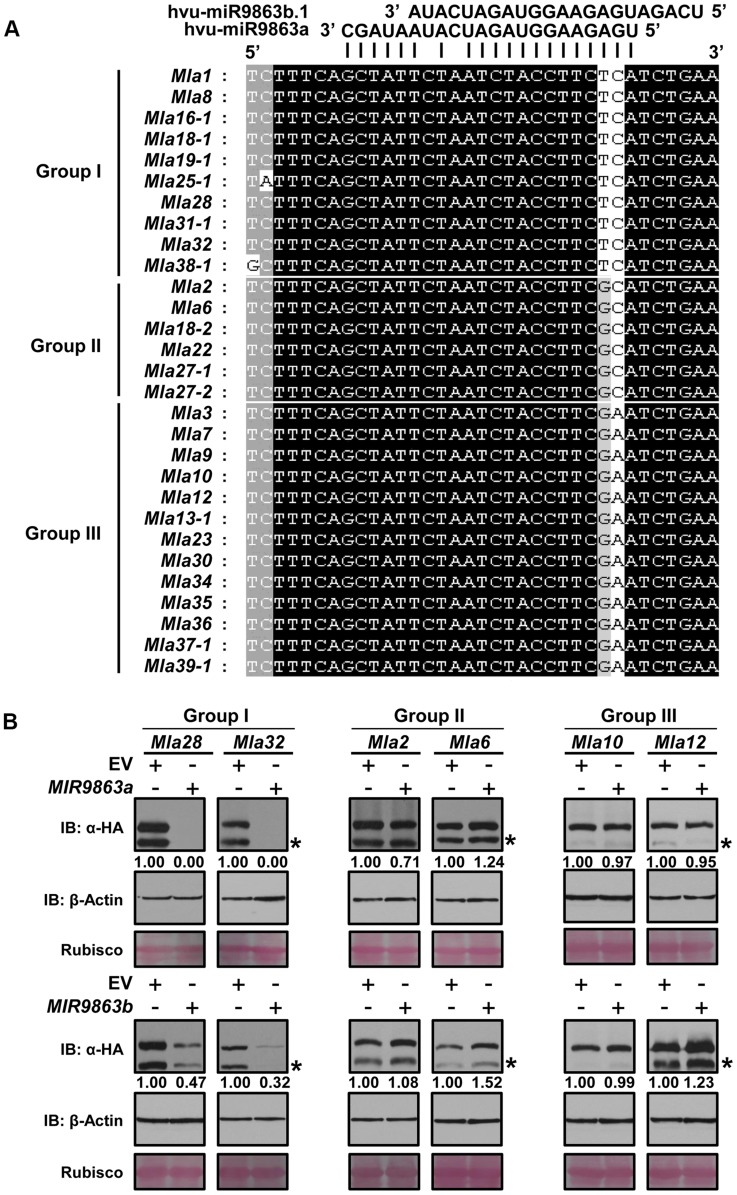
miR9863a and miR9863b.1/b.2 specifically regulate group I *Mla* alleles. (**A**) *Mla* alleles are classified into three groups according to the SNP haplotype in the miR9863 binding site. The two SNPs differ among *Mla* groups are highlighted. (**B**) *Mla* alleles of group I, but not group II and III, are regulated by miR9863a and miR9863b.1/b.2. *Mla* genes of group I (*Mla28*, *Mla32*), group II (*Mla2*, *Mla6*) and group III (*Mla10*, *Mla12*) were respectively co-expressed with either *MIR9863a* (upper panels) or *MIR9863b* (lower panels) in *N. benthamiana* as described in **Fig. 2**. Protein levels of MLA or actin were determined by immunoblotting with an anti-HA or anti-actin antibody; Rubisco was included as a loading control. The asterisks indicate non-specific signals.

Cloned *Mla* alleles encode highly sequence related CNL-subtype NLR receptors [48], [59], [75]–[78]. Sequence alignment using 29 *Mla* short sequences covering only the miR9863 target site shows that these *Mla* sequences are almost identical except for 1 or 2 adjacent nucleotides (Fig. 3A). These two adjacent nucleotides were predicted to complement with the 2^nd^–3^rd^ nt of miR9863a/b.2 or the 7^th^–8^th^ nt of miR9863b.1, respectively. Based on these SNP haplotypes, 29 *Mla* alleles were classified into three groups, group I, II and III (Fig. 3A). To determine whether there are differences in miR9863 regulation on different *Mla* groups, we randomly selected from each group two *Mla* genes, i.e., *Mla28* and *Mla32* from group I, *Mla2* and *Mla6* from group II, and *Mla10* and *Mla12* from group III, and each of them was co-expressed with precursor *MIR9863a* or *MIR9863b* in *N. benthamiana*, respectively (Fig. 1B, Fig. 3B). Western analysis revealed that the accumulation of MLA28 and MLA32 (group I) was fully blocked by expression of *MIR9863a* and partially blocked by expression of *MIR9863b* (Fig. 3B, left panels). By contrast, the accumulation of MLA2 and MLA6 (group II, Fig. 3B middle panel), or MLA10 and MLA12 (group III, Fig. 3B right panel) were not affected by expression of *MIR9863a* or *MIR9863b*, compared to the EV control. Together, these data strongly suggest that only group I *Mla* members are regulated by miR9863a and miR9863b.1/b.2 *in planta*.

**Figure 4:**
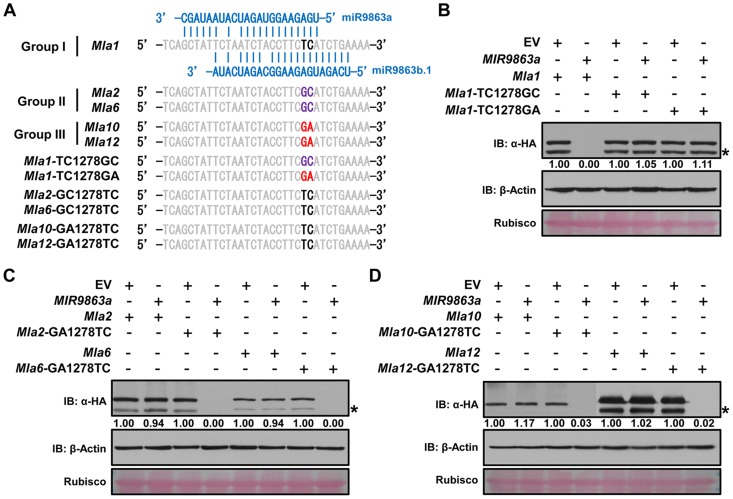
SNP variations in *Mla* alleles dictate miR9863 specificity. (**A**) Alignment of miR9863 target site of WT and mutated *Mla* alleles. SNPs in bold font and color-coded represent *Mla* type of group I (black), II (magenta) or III (red). Nucleotide substitutions TC1278GC or TC1278GA were introduced into *Mla1*; GC1278TC into *Mla2* and *Mla6*, and GA1278TC into *Mla10* and *Mla12*. (**B**) to (**D**) Determination of miR9863a regulation specificity on WT or mutated *Mla* alleles from different groups. Using transient gene expression assays in *N. benthamiana*, *Hvu-MIR9863a* was respectively co-expressed with group I *Mla1* alleles (B), or group II *Mla2* and *Mla6* alleles (C), or group III *Mla10* and *Mla12* alleles (D). The protein accumulation levels of indicated *Mla* isoforms and actin were determined by immunoblotting. The asterisks indicate non-specific signals.

To further verify the above finding that 1–2 SNPs in the *Mla* allele miR9863-binding site dictate miRNA specificity, we conducted targeted mutagenesis at these nt positions for each individual *Mla* from groups I, II, or III (Fig. 4A). First, we replaced the ‘TC’ (nt at 1278 and 1279) in *Mla1* (from group I) with ‘GC’ or ‘GA’ to mimic the SNP haplotype of group II or III *Mla*, and reciprocally we replaced ‘GC’ in *Mla2* and *Mla6*, as well as ‘GA’ in *Mla10* and *Mla12* to ‘TC’ to mimic the SNP haplotype of group I *Mla* (Fig. 4A). Upon co-expression of *MIR9863a* with WT *Mla1* or two *Mla1* variants (*Mla1*-TC1278GC, *Mla1*-TC1278GA), we could not detect by Western analysis any WT MLA1 accumulation, but the levels of two MLA1 variants are comparable to that in the EV control (Fig. 4B), suggesting loss of miR9863a effects on mutated *Mla1* genes. In contrast, when *MIR9863a* was co-expressed with WT *Mla2* and *Mla6*, or the respective *Mla* variants (Fig. 4C), as well as with WT *Mla10* and *Mla12*, or their variants (Fig. 4D), we detected the expression of the WT MLAs similar to EV control, but no accumulation was detected for all mutant variants, compared to the EV control (Fig. 4C and 4D). This indicates a gain of function for miR9863a for *Mla* variant genes that mimic the group I *Mla* SNP haplotype. We obtained similar gain of function results for *MIR9863b* (S7 Figure). These results suggest that miR9863 targeting specificity on *Mla* alleles is largely determined by the two SNP compositions in the miR9863-binding site of each *Mla*.

**Figure 5:**
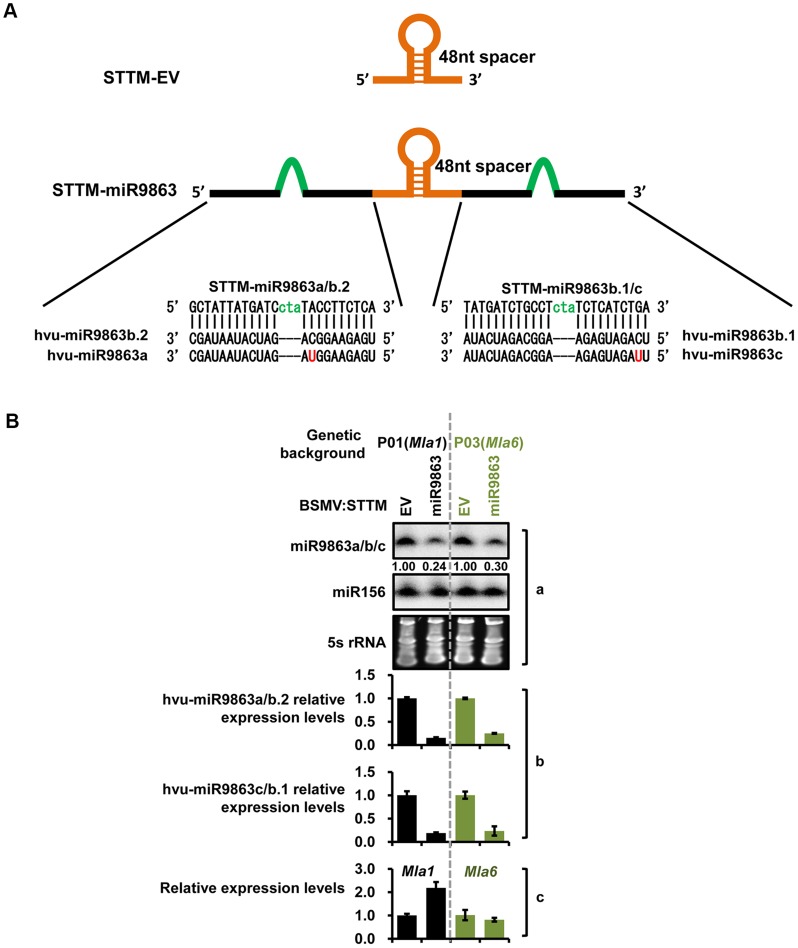
BSMV-VIGS silencing of miR9863 leads to increased transcript level of *Mla1* but not *Mla6* in barley. (**A**) Schematic representation of STTM-EV and STTM-miR9863 fragments used for the construction of pCaBS-γSTTM-EV and pCaBS-γSTTM-miR9863 for BSMV-mediated silencing (see Materials and Methods). (**B**) Comparison of expression levels of miR9863 members and *Mla* in BSMV infected barley plants. Pallas near-isogenic lines P01 (*Mla1*) and P03 (*Mla6*) were infected by BSMV harboring STTM-EV or STTM-miR9863 constructs, and systemic leaves with typical BSMV symptoms were collected for analysis. The level of miR9863 members was analyzed by RNA gel blot using miR156 as a loading control (panel a), or by qRT-PCR normalized against *U6* (panel b). *Mla1* and *Mla6* expression levels were quantified by qRT-PCR normalized against *actin* (panel c).

To further confirm miRNA-*Mla* specificity in barley, we conducted virus-induced gene silencing (VIGS) to knock-down expression of the miR9863 family in barley (Fig. 5). We first optimized a VIGS system by coupling a modified *Barley stripe mosaic virus* (BSMV)-induced gene silencing system with the expression of a short tandem target mimic (STTM), i.e., STTM-miR9863, designed to bind all barley miR9863 members (Fig. 5A) [79], [80] (see Materials and Methods). BSMV viral particles harboring the STTM-miR9863 structure were obtained from *N. benthamiana* and used to infect the near-isogenic barley line P01 (*Mla1*) or P03 (*Mla6*) (Fig. 5B, left and right half). As expected, we observed significantly reduced miR9863 accumulation in both P01 and P03 by BSMV: STTM-miR9863, compared to the BSMV-STTM-EV control by RNA blot analysis, while the accumulation of the non-related miR156 was not affected in both lines (Fig. 5B, panel a). Further qRT-PCR analyses validated the reduction of miR9863a/b.2 or miR9863c/b.1 in both lines infected with BSMV: STTM-miR9863, compared to BSMV-STTM-EV (Fig. 5B, panel b). Importantly, the reduced level of these miRNAs was inversely correlated with the increased *Mla1* transcript level in P01, in contrast to the unchanged *Mla6* transcript level in P03 (Fig. 5B, panel c). These data further verified that miR9863 family members specifically regulate group I *Mla* alleles. In summary, our results demonstrate that only a subset of *Mla* alleles are regulated by miR9863a and miR9863b.1/b.2, and two SNPs in the miR9863-binding site of *Mla* dictate miR9863 specificity.

### miRNA-directed phasiRNAs are critical for miR9863 regulation of *Mla*

**Figure 6:**
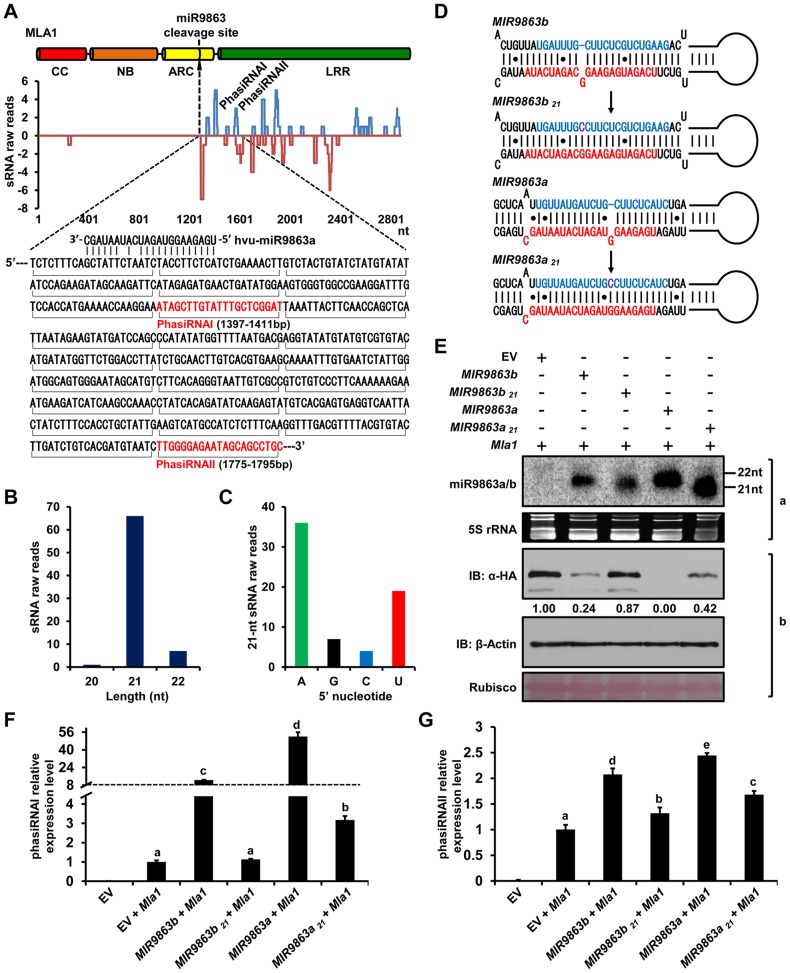
22-nt miR9863s trigger the production of phasiRNAs with *Mla1* transcripts. (**A**) Alignment of *Mla1* sequence with 21-nt phased phasiRNAs. Raw reads of barley 21-nt phasiRNAs (y axis) obtained from deep sequencing were mapped to the sense strand (blue lines above the x axis) or antisense strand (red lines below the x axis) of the *Mla1* sequence. *Mla1* sequence adjacent to the miR9863 cleavage site was shown below the plot, and horizontal brackets below the sequence indicate 21-nt phasiRNAs, of which two most abundant ones (phasiRNAI and phasiRNAII) are indicated in the plot and in the sequence (in red). (**B**) Length of *Mla1* phasiRNAs (x axis) was plotted to their raw reads value (y axis) obtained by sRNA deep sequencing in barley. (**C**) 5′-terminal nucleotide bias (x axis) of *Mla1* phasiRNAs determined by raw reads (y axis) obtained by sRNA deep sequencing in barley. (**D**) Predicted secondary structure of precursors for expressing natural 22-nt miRNAs (*MIR9863b*, *MIR9863a*) and mutated 21-nt miRNAs (*MIR9863b*_21_ and *MIR9863a*_21_). (**E**) miR9863b.1/b.2 and miR9863a of 22-nt regulate *Mla1* with higher efficiency than the mutated miRNAs of 21-nt. The mature miRNAs were detected by RNA gel blotting (panel a) with the mixture of [γ-^32^P]ATP labeled probes for miR9863a and miR9863b.1 (see supplemental **Table 2** online), and 5S rRNA was employed as a loading control. MLA1 and actin protein levels were determined by immunoblotting (panel b), and rubisco is shown as loading control. (**F**) to (**G**) miR9863b.1/b.2 and miR9863a of 22-nt trigger the biogenesis of phasiRNAs with *Mla1*. *Mla1* was co-expressed with EV or indicated miR9863 precursor in *N. benthamiana*, and the relative levels of phasiRNAI (F) and phasiRNAII (G) were measured by stem-loop quantitative RT-PCR (qRT-PCR) and normalized to *U6* level. Letters above the bars (a-e) represent groups with significant differences [*p*<0.05, Tukey's honest significant difference (HSD) test].

Apart from many *Bgh*-responsive miRNA families in the sRNA libraries derived from infected barley leaf tissue (above, and see Materials and Methods), we identified additional sRNAs that perfectly match the *Mla1* sense and antisense strand and are aligned downstream of the miR9863 target site (Fig. 6A, upper panel). These sRNAs are predominantly 21-nt in length (Fig. 6B) and biased for adenosine (A, ∼35%) and uridine (U, ∼20%) at the 5′-end position (Fig. 6C). Of the two abundant sRNAs at the 5′-proximal, one starts at 126 bp downstream of the miR9863a cleavage site (i.e., 1397 bp in *Mla1*), which corresponds to the 7^th^ 21-nt register from the cleavage site; and the other starts at 504 bp downstream of the miR9863a cleavage site and corresponds to the 25^th^ 21-nt register from the cleavage site (Fig. 6A, lower panel). These sRNAs are likely phasiRNAs [43] derived from the *Mla1* transcripts, thus, for convenience of further analysis these two 21-nt register siRNAs are designated as phasiRNAI and phasiRNAII, respectively. To understand whether the biogenesis of these phasiRNAs are directly linked to miRNA action on its target, we co-expressed *MIR9863b* or *MIR9863a* with *Mla1* in the heterologous *N. benthamiana* and then quantified the expression level of phasiRNAI and phasiRNAII (Fig. 6D, 6F and 6G). We found that phasiRNAI was significantly increased to ∼8 or 45 fold (Fig. 6F, bars 3 and 5), and phasiRNAII was increased to ∼2 to 2.5 fold (Fig. 6G, bars 3 and 5), compared to EV co-expressed with *Mla1* (Fig. 6F and 6G, bar 2). These results indicate that miR9863b.1/b.2 and miR9863a trigger the biogenesis of phasiRNAs with *Mla1* in *N. benthamiana*, and are consistent with our sRNA deep sequencing data obtained from barley.

Previous studies have demonstrated that miRNAs of 22-nt, rather than 21-nt, trigger secondary siRNA [38], [39], [43]–[45]. To test whether this also applies to miR9863 for *Mla-*dependent secondary siRNA production, we engineered the precursor of *MIR9863b* or *MIR9863a* by adding a cytosine in the miR9863* sequence to remove the asymmetric structure, resulting in *MIR9863b_21_* and *MIR9863a_21_* (Fig. 6D). This construct is predicted to produce 21-nt miRNAs that retain target-binding and cleavage activity [39], [44], [45]. Indeed, upon co-expression of each of these precursors with *Mla1* in *N. benthamiana*, we detected by RNA gel blotting that *MIR9863b_21_* and *MIR9863a_21_* predominantly generated 21-nt miRNAs while the natural *MIRNA* precursors produced 22-nt miRNAs (Fig. 6E, panel a). Significantly, compared to the natural *MIRNA* precursors, *MIR9863b_21_* and *MIR9863a_21_* less effectively repressed MLA1 accumulation (Fig. 6E, panel b). Interestingly, we also observed marked reduction of secondary siRNAs, representing by phasiRNAI and phasiRNAII, in samples expressing mutated precursors (Fig. 6F and 6G, bar 4 and 6), compared to the WT precursors (Fig. 6F and 6G, bar 3 and 5). Together, these data suggest that the ineffective *Mla1* regulation by the 21-nt-long miRNAs is partially due to the reduced level of secondary siRNAs. This was further supported by co-expressing *MIR9863a* or *MIR9863a_21_* with a short *Mla1* fragment encoding only the ARC domain fused with the mYFP-3HA tandem tag (S8 Figure). In samples expressing miR9863a_21_ of 21-nt, we detected almost no phasiRNAs by RNA gel blotting, compared to the substantial phasiRNA accumulation in samples expressing 22-nt natural miR9863a (S8B Figure, panel a). Similarly, the level of phasiRNAs was inversely correlated with the abundance of MLA1_ARC fusion protein (S8B Figure, panel b). In summary, these results demonstrate that 22-nt natural miR9863s are critical for the biogenesis of phasiRNAs of 21-nt in barley and *N. benthamiana*, and that these secondary phasiRNAs are important for effective miR9863 regulation of *Mla* target genes.

### miR9863-mediated *Mla* regulation likely requires AGO1

The sorting of miRNAs to different AGO proteins is complex and influenced by several factors [24], [25], for example, the 5′ nucleotide identity and the length of miRNAs, and miRNA duplex structure. Arabidopsis AGO1 has been shown to associate with miRNAs with 5′ U [81] and to trigger the production of secondary siRNA when binding 22-nt miRNAs [25]. AGO genes have not yet been formerly annotated in barley genome although some sequences share high similarity with *AtAGO1*. To test whether *AGO1* might be involved in miR9863-mediated regulation of *Mla*, we took advantage of the *Tobacco rattle virus* (TRV) mediated-VIGS to knock-down *AGO1* in *N. benthamiana* using *AGO4* as a control (S9 Figure). Arabidopsis *AGO4* was shown to associate primarily with 24-nt siRNAs [81], [82]. As a technical control, the TRV-VIGS silencing of *PHYTOENE DESATURASE* (*NbPDS*) worked effectively, resulting in clear photobleaching phenotype on the upper systemic leaves (S9C Figure). Both *NbAGO1* and *NbAGO4* possess two alleles each, i.e., *NbAGO1-1* and *NbAGO1-2*, *NbAGO4-1* and *NbAGO4-2*, and these alleles share high sequence similarity [83]. Indeed, in our TRV-VIGS assays we observed co-suppression of both alleles of *NbAGO1* or *NbAGO4* when either one of the alleles was targeted for silencing (S9B Figure, top and middle panel). Interestingly, upon co-expression of *Mla1* with *MIR9863b* or *MIR9863a*, MLA1 accumulation was similarly suppressed in TRV: 00 treated plants or *NbAGO4*-silenced plants (S9D Figure, 1^st^, 4^th^ and 5^th^ column). This is in contrast to *NbAGO1*-silenced plants, where MLA1 accumulation was unaffected, as compared to the TRV: 00 controls (S9D Figure, 2^nd^ and 3^rd^ column); the expression level of miR9863b or miR9863a was similar in all samples, however. Together, these data indicate that *Nb*AGO1 rather than *Nb*AGO4 is required for miR9863-mediated regulation of *Mla1* in *N. benthamiana*.

### Translational repression may contribute to miR9863-mediated regulation of *Mla*

**Figure 7:**
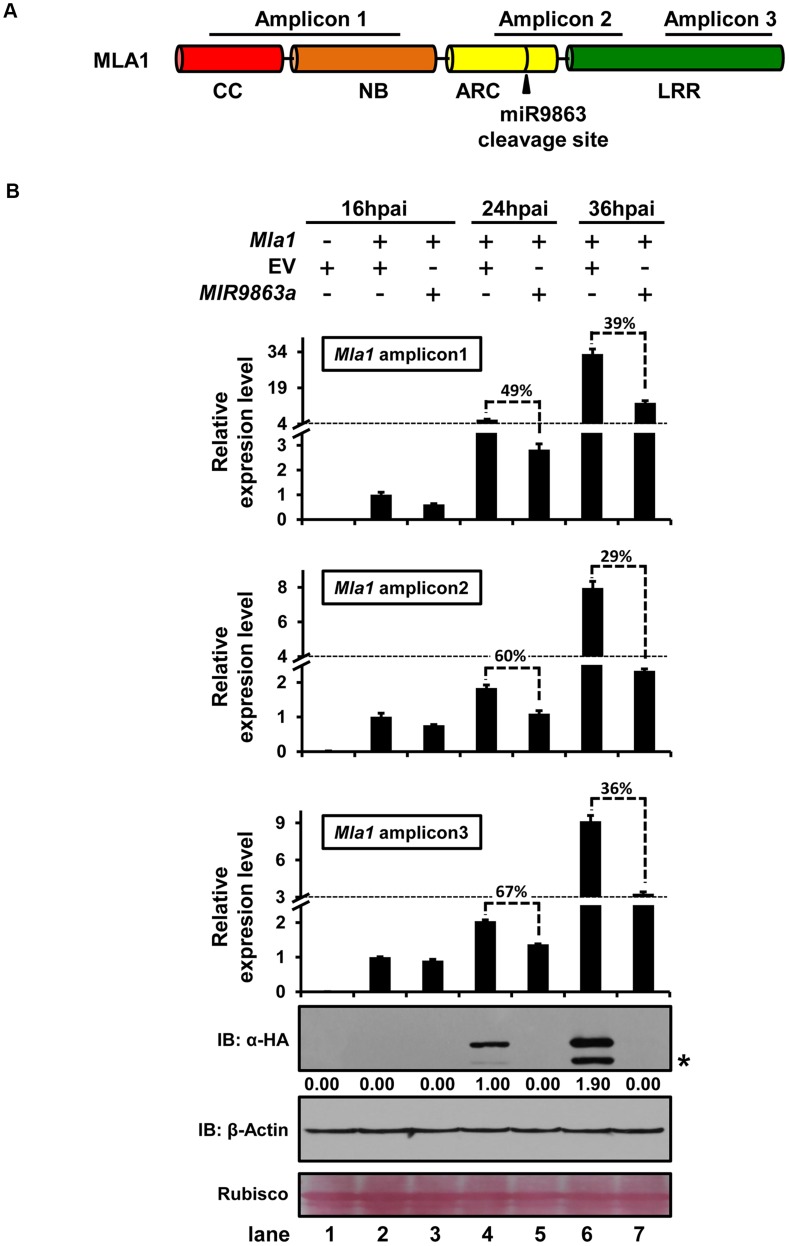
miR9863a regulates *Mla1* mRNA and MLA1 protein in an uncoupled manner. (**A**) Schematic representation of *Mla1-*encoded domain structure and positions of amplicons for qRT-PCR quantification of *Mla1* mRNA level. Amplicon 1, 2 or 3 positions at 5′, over or 3′ of the miR9863 cleavage site, respectively. (**B**) Quantification of *Mla1* transcript and protein level at different time points post co-expression of *Mla1*-3HA with *MIR9863a* in *N. benthamiana*. The relative levels of three *Mla1* amplicons were analyzed for samples of three time points, 16 and 24 and 36 hours-post-Agro-infiltration (hpai), by qRT-PCR quantification. For each amplicon, relative level was shown as fold-changes compared to the level at 16 hpai after first normalized to *actin* for each time point. The percentage above the bars indicates the remaining level of the amplicons relative to that of the EV control. Samples used for protein quantification were collected at 2 hrs after taking samples for mRNA quantification allowing sufficient time for protein translations. Western blots shown are exposed for more than 2 hrs. The asterisks indicate non-specific signals. All the experiments were performed at least twice with similar results.

Our data so far have showed that miR9863-directed *Mla* transcript cleavage and degradation play a role in the regulation of *Mla*. To examine whether translational inhibition directed by miR9863 may contribute to MLA suppression, again we co-expressed *MIR9863a* with *Mla1* in *N. benthamiana* and then quantified *Mla1* transcripts in parallel with MLA1 protein levels (Fig. 7). We monitored the level of three *Mla1* amplicons (amplicon 1, *Mla1*_407–783_; amplicon 2, *Mla1*_1228–1582_; amplicon 3 *Mla1*_2465–2621_), positioned respectively upstream, over and downstream of the miR9863a cleavage site to reflect the levels of transcription, cleavage and cleavage-triggered decay of *Mla1* transcripts (Fig. 7A) [84], at 16, 24, and 36 hours-post-Agro-infiltration (hpai). The corresponding MLA1 protein level was determined in samples collected 2 hrs later for each time point, allowing sufficient time for translation (Fig. 7B) [84]. At 16 hpai, we detected a basal level of the three *Mla1* amplicons, but no accumulation of MLA1 protein (Fig. 7B, lanes 2 and 3). Later, at 24 hpai, we detected increased accumulation of *Mla1* amplicons, as well as the presence of MLA1 protein in samples co-expressing the EV control (Fig. 7B, lane 4). However, the levels of the three *Mla1* amplicons in samples co-expressing *MIR9863a* were 49–67% of that in EV control and MLA1 protein remained undetectable in the same samples (Fig. 7B, lane 5). At 36 hpai, *Mla1* amplicons and MLA1 protein further increased in the EV control (Fig. 7B, lane 6), but the three *Mla1* amplicons in *MIR9863a*-expressing samples were only 29–39% of the EV control (Fig. 7B, lane 7 vs. lane 6). It should be noted that these *Mla1* transcript levels in the *MIR9863a*-expressing samples at 36 hpai are higher than those in the EV controls at 24 hpai (Fig. 7B, lane 7 vs. lane 4), yet MLA1 protein accumulation was not observed at 36 hpai, even though MLA1 protein was detected in the EV control at 24 hpai (Fig. 7B, lane 7 vs. lane 4). These findings suggest that in *N. benthamiana Mla1* transcript levels have been uncoupled from accumulation of MLA1 protein in samples that *Mla1* was co-expressed with miR9863, indicating apart from miR9863a-mediated transcript cleavage and decay, miR9863a-directed translational repression also plays a role in the regulation of *Mla1* in this heterologous expression system.

### Overexpression of miR9863 members attenuates MLA-triggered disease resistance and cell-death signaling

**Figure 8:**
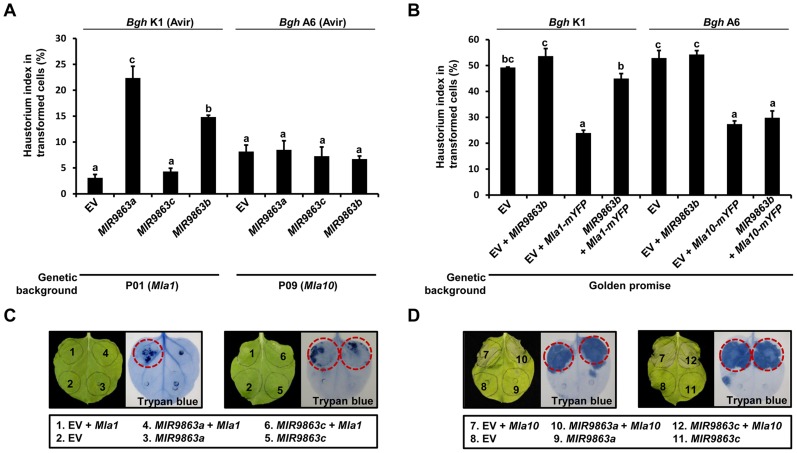
Overexpression of *MIR9863a* and *MIR9863b* attenuates *Mla1-*triggered disease resistance and cell-death signaling. (**A**) Transient gene expression of *MIR9863a, MIR9863c* and *MIR9863b* in barley near-isogenic line containing *Mla1* or *Mla10*. Empty vector or plasmids expressing indicated *MIRNA* precursors were delivered into leaf epidermal cells of indicated barley lines through particle bombardment, and spores of indicated avirulence *Bgh* isolates were inoculated 36 hrs post bombardment. Haustorial index shown are the percentage of cells that developed fungal haustoria in all transformed cells. (**B**) Transient gene expression assays in Golden promise (GP), a barley line susceptible to both *Bgh* K1 and *Bgh* A6. Indicated construct expressing *MIR9863b* was delivered alone or with *Mla1*-mYFP or *Mla10*-mYFP construct into GP leaf epidermal cells, and spores of *Bgh* K1 or *Bgh* A6 were inoculated at 4 hrs post bombardment. Error bars in (**A**) and (**B**) represent standard error (SE) from at least three replicates, and letters (a–c) above the bars represent groups with significant differences [p<0.05, Tukey's honest significant difference (HSD) test]. (**C**) to (**D**) Analysis of cell-death induction upon co-expressing *Mla1* or *Mla10* with *MIR9863a* and *MIR9863c*. The indicated constructs were expressed in *N. benthamiana* leaves by Agro-infiltration, and cell death was visualized by trypan blue staining at 48 hours-post-Agro-infiltration (hpai). Red circle indicates visible cell-death phenotype.

To investigate the role of miR9863 members in MLA-mediated disease resistance to *Bgh*, we used a well-established single-cell transient assay [77], [85]. Plasmid constructs of miRNA precursor are co-delivered with a β-glucuronidase (GUS) reporter into barley epidermal cells by particle bombardment, and then the haustorial index (HI%) is scored to assess the frequency of fungal haustoria in transformed cells upon fungal inoculation (Fig. 8A and 8B). First, we respectively delivered plasmids of EV, *MIR9863a*, *MIR9863c*, or *MIR9863b* into near-isogenic barley line P01 (*Mla1*) and then inoculated conidiospores of *Bgh* K1 (*AVR_a1_*) to activate MLA1-mediated immune responses (Fig. 8A, left panel). We observed a low HI% of ∼3% in leaves receiving EV control, likely due to MLA1-triggered immunity against *Bgh* K1 (Fig. 8A, bar 1), whereas in leaves expressing *MIR9863a* or *MIR9863b*, we observed significantly increased HI% to ∼22% or 15% (Fig. 8A, bars 2 and 4), indicating MLA1-triggered immunity was compromised by the expression of miR9863a or miR9863b.1/b.2. The HI% was similar in P01 leaves expressing *MIR9863c* to that in the EV control (Fig. 8A, bar 3), consistent with that miR9863c does not interact with the *Mla1* allele (Fig. 2C). Further, plasmids of *MIR9863a*, *MIR9863c* and *MIR9863b* were respectively delivered into leaves of near-isogenic barley line P09 (*Mla10*) and inoculated with *Bgh* isolate A6 (*AVR_a10_*) (Fig. 8A, right panel), and we observed comparable HI% of ∼6% to 7% in leaves expressing EV or respective miR9863 precursors (Fig. 8A, bars 5 to 8), suggesting that expression of any miR9863 members had no effect on *Mla10*-mediated disease resistance to *Bgh*, consistent with that *Mla* members of group III are not miR9863 targets (Fig. 3B). Taken together, overexpression of miR9863a and miR9863b.1/b.2 specifically attenuates MLA1 but not MLA10-mediated disease resistance against *Bgh* in barley.

To exclude non-specific effects on barley defense responses due to miRNA overexpression, for example basal immunity, we delivered EV alone or together with *MIR9863b* into leaves of Golden Promise (GP) that is susceptible to both *Bgh* isolates K1 and A6 (Fig. 8B). HI% scored on GP leaves expressing EV control, or co-expressing EV with *MIR9863b*, was ∼50% upon inoculation with *Bgh* K1 or A6, suggesting that expression of miR9863b.1/b.2 does not interfere with basal defense to *Bgh* (Fig. 8B, bars 1 and 2, bars 5 and 6). Furthermore, functional *Mla1-mYFP* or *Mla10-mYFP* fusion [52] was co-delivered into GP leaves with either EV or *MIR9863b*, respectively (Fig. 8B, bars 3 and 4, 7 and 8). *Mla1*-*mYFP* co-expression with EV or *MIR9863b* resulted in contrasting HI% for *Bgh* K1 (*AVR_a1_*), ∼24% vs. ∼45% (Fig. 8B, bars 3 and 4), indicating again that MLA1-mediated responses against *Bgh* K1 was compromised by miR9863b.1/b.2 expression; in contrast, *Mla10-mYFP* co-expression with EV or *MIR9863b* resulted in similar HI% of ∼25% to *Bgh* A6 (*AVR_a10_*), thus retained MLA10-triggered disease resistance to *Bgh* A6 (Fig. 8B, bars 7 and 8), consistent with the finding that *Mla10* is not a miR9863 target (Fig. 3, Fig. 4).

MLA triggered cell-death is conserved in dicot plants [56], [57], thus, we reasoned that miR9863 targeting MLA may also affect this MLA activity in dicots. To test this possibility, we co-expressed *MIR9863a* or *MIR9863c* respectively with *Mla1* (Fig. 8C), or with *Mla10* (Fig. 8D), in *N. benthamiana*. *Mla1* co-expression with EV effectively triggered cell death detected by trypan blue staining at 36∼48 hpai, whereas this cell-death was completely abolished in *Mla1* co-expression with *MIR9863a* (Fig. 8C, left panel), but not with *MIR9863c* (Fig. 8C, right panel). Alternatively, *Mla10* co-expression with EV triggered strong cell-death that was unaffected by expressing either *MIR9863a* or *MIR9863c* (Fig. 8D, left and right). These results indicate that miR9863a retains regulation specificity in suppressing MLA1, but not MLA10, to induce cell-death in dicot plants.

### Expression of *Mla1*, miR9863 members and phasiRNAs during *Bgh* infection

To further understand the regulation of miR9863 on *Mla* target in the context of *Bgh* infections, we analyzed the expression of *Mla1*, miR9863 members and two phasiRNAs during incompatible barley-*Bgh* interactions (S10 Figure). A barley transgenic line expressing functional *Mla1-*HA fusion driven by *Mla1* native promoter [61] was inoculated with *Bgh* K1 (*AVR_a1_*) and gene expression was monitored up to 72 hrs post inoculation (hpi). *Mla1* transcripts were induced at 16 hpi by *Bgh* K1 and reached the highest level at 24 hpi (S10A Figure). Similar results were previously reported for the expression of *Mla6* and *Mla13* during incompatible interactions [76], [86]. During the same period of time from 16 to 24 hpi, we also detected by stem-loop qRT-PCR the increased expression of miR9863a/b.2 and miR9863c/b.1, as well as phasiRNAI and phasiRNAII (S10B and C Figure, Fig. 6A), noting that here we cannot distinguish miR9863a from miR9863b.2, or miR9863c from miR9863b.1, due to only one nt difference. Accumulation of *Mla1* transcripts dropped from ∼4.0 fold at 24 hpi to ∼1.6 fold at 48 to 72 hpi (S10A Figure), and inversely, the expression level of miR9863c/b.1, and the two phasiRNAs was increased and reached to the highest level at 48 hpi and sustained at high level up to 72 hpi (S10 B and C Figure). Interestingly, miR9863a/b.2 expression was induced earlier (at 16 hpi) than miR9863c/b.1 (at 24 hpi), and the former maintained at similar level while the latter continued to increase up to 72 hpi (S10B Figure). These data confirm the induction of *Mla* gene expression in the early phase (16∼24 hpi) during incompatible interactions [76], [86] and suggest that, in the later phase of the interactions, the decreased *Mla1* expression level is coupled to the induced or sustained expression of miR9863s and the phasiRNAs.

## Discussion

We present here a regulatory network comprising miR9863 family members and the derived secondary phasiRNAs that target *Mla* alleles during barley-*Bgh* interactions. This post-transcriptional mechanism appears to utilize more than one mode of action in miRNA-mediated target regulation, i.e., *Mla* mRNA cleavage, shown in both barley and *N. benthamiana* (Fig. 1 and S4 Figure), and translational inhibition, shown in *N. benthamiana* (Fig. 7). Overexpression of miR9863 members attenuate group I MLA-mediated disease resistance to *Bgh* in barley and its heterologous cell-death response in *N. benthamiana* (Fig. 8). This dedicated miR9863 function is likely related to the specificity defined by the miR9863-*Mla* pair and dictated by SNP variations in mature miR9863s and the miRNA-targeting site in unique *Mla* alleles (Figs. 1, 3 and 4; S6 and S7 Figures). Furthermore, BSMV-mediated silencing of miR9863 in barley led to the up-regulation of *Mla1*, but not *Mla6* transcripts (Fig. 5), and time-course expression analysis revealed that the down-regulation of *Mla1* transcript accumulation is inversely correlated with the up-regulation of miR9863s and phasiRNAs in the late phase of barley-*Bgh* incompatible interactions (S10 Figure). Interestingly, for many *Mla* alleles, there also exists a correlation between their derepression of miR9863 regulation with their dependence on RAR1, a cochaperone required for accumulation of a subset NLR receptors (S11 Figure) (see below). Therefore, we propose that the miR9863 family plays a key role in targeting *Mla* alleles and in dampening the disease resistance and associated cell-death triggered by these NLR proteins.

### miR9863 family target *NLR* genes in *Triticeae* species

Although miRNAs regulating NLR-encoding genes have recently been demonstrated in a wide variety of dicot species and perennial woody plants [38]–[41], [87], convincing evidence for similar regulatory mechanisms have been scarce in well characterized monocot species, for example, rice and maize. Based on earlier work, it was postulated that *NLR* genes in *Poaceae* species were not regulated by endogenous miRNA and siRNA regulatory networks [43]. However, regulation of barley *Mla* alleles by the miR9863 family demonstrates that these mechanisms are present in *Poaceae* species. Moreover, previous small RNA sequencing studies have identified various members of the miR9863 family [63]–[66], [88], suggesting functional conservation in barley and wheat. Through data mining and mRNA expression analyses, we show that the miR9863 family appears to be absent in dicot species *N. benthamiana* and *A. thaliana*, as well as monocots rice (*O. sativa*), maize (*Z. mays*) and *B. distachyon*, at least at the depth of coverage in current miRNA databases (S2 and S3 Figures). Although some putative sorghum small RNAs were found sequentially matching miR9863a, it appears that the miR9863 family is mainly expressed in barley and wheat, suggesting that miR9863 family might be *Triticeae*-specific. Since functional *Mla* homologs and orthologs exist in wheat, it will be interesting to know whether miR9863 members regulate these genes or other *NLR* genes as well (S1 Figure) [89], [90].

### SNPs in miRNA and target miRNA-binding sites determine regulation specificity

Unlike animal miRNAs, plant miRNAs largely depend on their high sequence complementarity to target mRNAs for post-transcriptional gene regulation [9]. Indeed, Arabidopsis and rice genome-wide assessments of sequence variation have revealed lower levels of nucleotide variation and divergence in miRNAs and target binding sites than in their flanking sequences [73], [91], leading to hypothesis that strong purifying selection plays an important role in the interaction between plant miRNAs and their target binding sites. Somewhat surprisingly, similar findings were also reported in humans [92], [93]. Nevertheless, few SNP variations in miRNAs and their target binding sites have been identified in plants [73], [91], [94], [95], and some of them have been shown to contribute to phenotypic variations in Arabidopsis [95].

Our analyses of miR9863s and *Mla* target sequences have uncovered the role of miR9863 family SNPs in determining target specificity and potentially phenotypic variations (Figs. 1, 2 and 3; S6 Figure). The miR9863a and miR9863b.2 differ by only one SNP at the 9^th^ nt position (Fig. 1A), however, they account for ∼60–80% difference in *Mla1* target protein accumulation upon coexpression in *N. benthamiana* (Fig. 2A, 2E; S6 Figure). Similarly, one SNP variation at 2^nd^ nt position in miR9863c and miR9863b.1 can fully dictate the regulation specificity on *Mla1* (Fig. 1A; Fig. 2B, 2C and 2E). In this context, it is worth noting that the *NLR*-targeting miR482/2118 superfamily contains at least 9 SNPs and 31 isoforms across diverse plant species [38], [40]. Since many isoforms of the miR482/2118 family are species or genera specific, it is possible that the SNP variations within members of this superfamily have evolved to allow specific isoforms to regulate species-specific *NLR* genes and other targets as well [40].

A single or two adjacent SNPs in *Mla* alleles can dictate the regulation specificity of the miRNA members on *Mla*, thus, group I *Mla* but not group II or III *Mla* are targets of miR9863 members (Figs. 3 and 4; S7 Figure). The above findings are supported not only by miRNA expression in the heterologous *N. benthamiana* system, but also by miRNA silencing in barley using BMSV-VIGS coupled with STTM technology (Figs. 4 and 5). Moreover, single cell transient expression assays in barley have demonstrated that overexpression of *MIR9863a* or *MIR9863b* specifically attenuated *Mla1*- but not *Mla10*-mediated disease resistance (Fig. 8A); similarly, miR9863a expression in *N*. *benthamiana* blocked *Mla1*- rather than *Mla10*-triggered cell death (Fig. 8C and 8D). Therefore, miR9863 variants paired with their respective *Mla* alleles supports the notion that SNP variations in the miRNA and the target binding site are important in determining regulation specificity as well as in phenotypic variations in plants. We believe that this is particularly crucial for the control of the large *NLR* gene family, to allow for balancing the fitness cost and effectively coping with the fast evolving pathogen isolates.

### Evolution of *Mla* alleles with respect to miR9863 regulation

Although the *Mla* locus encodes highly related alleles apart from other dissimilar *RGH* families [48], [77], [96], only a subset of these alleles are regulated by three out of four miR9863 family members presented here. We postulate that specificity between *Mla* and miR9863 is determined by SNP variation(s) in members among miR9863 family as well as in the binding site of target *Mla* alleles (see above). Nonetheless, the evolutionary and functional relevance of *Mla*-miRNA regulation remains unclear. To shade light on this aspect, we utilized the cDNA sequences encoding full-length MLA, CC, NB-ARC or LRR domain to respectively construct a phylogeny tree of the 29 *Mla* alleles (S11 Figure). When each individual *Mla* is mapped in accordance with subgroup identity based on SNPs in the target site, i.e., group I, II and III (see Fig. 3A), we observed more similar architecture for the phylogeny tree derived from full-length or NB-ARC cDNA sequence than to the others (S11 Figure, A and C). Most interestingly, in the NB-ARC phylogeny tree, *Mla* alleles of group II and III comprise one more recent and distinct branch whereas group I *Mla* constitute a more complex pattern, indicating group II and III *Mla* are evolutionarily closer to each other than to group I *Mla* with regard to regulation by miR9863. Indeed, we show that this control only applies to group I *Mla* alleles. This is reminiscent of the proposed parallel evolutionary pathways for the *Mla* family, i.e., one branch contains *Mla1* (from group I) and the other branch contains *Mla6* and *Mla13* (from group II and III, respectively) [59]. Thus, we speculate that the evolution of the *Mla* family might be influenced by the adaptation of miR9863. In light of this, natural SNP variations might be fixed within the miR9863 binding site of group II and III *Mla*, which result in the deregulation of the miRNAs and subsequent formation of a *Mla* clade distinctive from group I *Mla* members.

Additional evidence to support evolutionary pathways differentiating group I *Mla* alleles from group II and III comes from the differential *Rar1*-dependency of *Mla* alleles. RAR1 is believed to act as a cochaperone and assist NB-LRR proteins of low accumulation level to reach threshold steady-state level for effective immunity [60], [61], [97], [98]. For example, MLA6 accumulates only one fourth the level of MLA1, and thus depends on RAR1 to maintain a threshold level to mediate disease resistance [61]. Interestingly enough, while *Mla1* from group I is *Rar1*-independent, *Mla* alleles from group II or III, such as *Mla6*, *Mla9*, *Mla12*, *Mla13*, *Mla22* and *Mla23*, excepting *Mla7*, are all *Rar1*-dependent [59], [99] (Fig. 3; S10 Figure). One would hypothesize that, in barley and barley powdery mildew interactions, *Mla*-miR9863 adaptations are likely fixed for *Mla* alleles whose expression level and protein accumulation are high, for example those of group I; whereas for *Mla* natural variants, like members of group II and III that are unstable or with lower accumulation level, the miR9863 regulation might be unnecessary and thus released. Nevertheless, how the evolution of *Mla* branches is coupled with miR9863 regulation as well as the cochaperone RAR1 requires future investigation.

### Implications from using the *N. benthamiana* expression system in dissecting miR9863 regulation of *Mla* alleles

In the present study, we leveraged the heterologous *N. benthamiana* expression system to investigate the regulation of barley miR9863 on its barley *NLR* targets. This system allows the testing of individual components in the absence of unknown confounding factors in the barley host. Indeed, despite the apparent absence of the miR9863 family in the dicotyledonous *N. benthamiana* and Arabidopsis (S3 Figure, and discussion above), transient expression of barley and wheat miR9863 progenitors or artificial miRNA precursors in *N. benthamiana* can generate respective mature miRNAs of the correct size (Fig. 2D; S2B Figure), suggesting that a conserved small RNA biogenesis machinery exists in both monocots and dicots. This is further corroborated by the observation of direct coexpression of 22-nt miR9863 members and *Mla1* in *N. benthamiana* which enhanced the production of 21-nt phasiRNAs, and that many of these miR9863 members were identified in sRNA libraries derived from *Mla1*-containing barley isogenic line (Fig. 6).

VIGS of *NbAGO1* in *N. benthamiana* resulted in the loss of miR9863a or miR9863b control of *Mla1.* This suggests that AGO1 may be functionally conserved in *N. benthamiana* and in barley (S9 Figure). This is consistent with previous observation that plant AGO1 has the capacity to bind 22-nt miRNAs and trigger the production of secondary siRNAs [25]. It would be interesting in future experiments to test whether in barley 22-nt miR9863 members are sorted into AGO1-containing complex where they direct *Mla* transcript cleavage.

Lastly, previous studies found that barley MLA1-triggered immunity against *Bgh* fungal isolate is fully retained in Arabidopsis and that MLA-triggered cell-death signaling is likely conserved in Arabidopsis and *N. benthamiana* [56]–[58]. Similarly, recent studies show that expression of some wheat *Pm* alleles, conferring resistance against *B. graminis* pathogens, also triggers cell-death responses in *N. benthamiana* [100], [101]. Interestingly, another study also demonstrates that interfamily transfer of the dual TIR-type *NLR* genes, RPS4/RRS1, from Arabidopsis into other Brassicaceae plants and Solanaceae species, confers broad-spectrum or isolate-specific disease resistance to fungal or bacterial pathogens [102]. These examples suggest that some plant NLR receptors may engage evolutionarily conserved downstream signaling components for triggering immune responses. Here, coexpression of miR9863a with *Mla1* or *Mla10* in *N. benthamiana* attenuates *Mla1*- but not *Mla10*-triggered cell-death responses (Fig. 8), together with above-mentioned retained miR9863 specificity on *Mla* alleles in *N. benthamiana*, might imply a conserved post-transcriptional regulatory machinery acting on heterologously expressed barley *Mla NLR* genes.

## Materials and Methods

### Plant and fungal materials

Barley (*Hordeum vulgare* L.), bread wheat (*Triticum aestivum* L.) and *Brachypodium distachyon* plants were grown in a growth chamber under a 16 hrs/8 hrs, 20°C/18°C day/night cycle, respectively, with 70% relative humidity; Rice (*O. sativa*) was maintained in a growth chamber under a 16 hrs/8 hrs, 28°C/26°C day/night cycle, respectively, with 90% relative humidity; Maize (*Z. mays*) was grown in a chamber at 22°C under a 16 hrs/8 hrs day/night cycle under 70% relative humidity; *N. benthamiana* and *A. thaliana* were grown in greenhouse at 24±1°C with a 16 hr light period.

*Blumeria graminis* f. sp. *hordei* (*Bgh*) isolates K1 (*AVR_a1_*, *vir_a6_*, *vir_a10_*, *vir_a12_*) and A6 (*AVR_a6_*, *AVR_a10_*, *AVR_a12_*, *vir_a1_*) were maintained on barley cultivar ‘I10’ (containing *Mla12*) and ‘P01’ (containing *Mla1*), respectively, and kept at 70% relative humidity, and a 16 hrs/8 hrs, 20°C/18°C day/night cycle.

### Small RNA isolation and deep sequencing

Seven-day-old barley leaves of barley isogenic line P01 were inoculated with *Bgh* A6 and K1 for 22 hrs and total RNAs were isolated using TRIzol solution (Invitrogen 15596-026) according to the manufacturer's instructions. Small RNAs of 18–30 nt were excised and isolated from 5 to 10 µg total RNAs electrophoresed on 15% polyacrylamide denaturing gel, and then were ligated with 5′ and 3′ adapters. The ligated small RNAs were used as templates for cDNA synthesis followed by PCR amplification. The obtained libraries were sequenced using the Solexa sequencing platform (BGI, Beijing).

### *MIRNA* precursor identification, secondary structure prediction, and cloning

The mature miRNAs sequences were used in a BLASTn search against the barley genome sequencing database (http://webblast.ipk-gatersleben.de/barley/) and bread wheat expressed sequence tag (EST) database (http://www.ncbi.nlm.nih.gov/). The secondary structure of flanking sequence around perfectly matched site was predicted using the RNA-folding program Mfold [103].

Primers for cloning of *MIR9863* precursors were designed according to the flanking sequences of the hairpin structures. Primers (J19/J20) for *hvu-MIR9863a* cloning were designed according to the sequence of *tae-MIR9863a*. *hvu-MIR9863b* was PCR-amplified using barley ‘Morex’ genomic DNA as template (primers, J15/J16); *tae-MIR9863* cluster containing *tae-MIR9863a* and *tae-MIR9863b* was amplified using bread wheat ‘Chancellor’ genomic DNA as template and primer J05/J06; *tae-MIR9863c* was amplified using bread wheat ‘Chancellor’ genomic DNA as template and primer J07/J08. All primer sequences are shown in supplemental **S2 Table**.

### Validation of miRNA cleavage site by 5′ RACE assay

RLM-RACE kit (TaKaRa, Code D315) was used for 5′ RACE according to the manufacturer's instruction. Total RNAs were isolated from 7-day-old leaves of barley line P01 infected with *Bgh* K1 spores, and mRNAs were enriched from 100 µg of total RNAs using the PolyATtract mRNA isolation kit (Promega, Z5210). The RNA Oligo adaptor was ligated to mRNAs without calf intestinal phosphatase treatment. For the first round PCR, the 5′ RACE Outer Primer J01 together with *Mla1* gene specific outer primer J03 were used. Nested PCR amplification was performed using the 5′ RACE Inner Primer J02 and *Mla1* specific inner primer J04.

### Plasmid constructions

Plasmids constructed in this study are based on several expression vectors previously described and listed in supplemental **S3 Table**.

CTAPi-GW-3HA was used as the transient expression vector for *Mla* genes in *N. benthamiana*, and derived plasmids were constructed using Gateway technology (Invitrogen) following the instructions of the manufacturer.

For construction of miRNAs overexpression constructs used in *N. benthamiana* expression assays, indicated *MIRNAs* precursor sequences were PCR amplified, double digested by KpnI/HindIII, and ligated into 35S-pKANNIBAL vector. Precursor *aMIR9863a*, *aMIR9863b.1* and *aMIR9863b.2* were constructed by replacing the miR173/miR173* duplex in *ath-MIR173* with amiR9863a/amiR9863a*, amiR9863b.1/amiR9863b.1* or amiR9863b.2/amiR9863b.2* (**S5 Figure**) using overlapping primers J35/J36/J37, J31/J32/J33 and J36/J37/J38, respectively.

pTRV2-derivatives used in TRV-VIGS analysis were constructed based on pTRV2-LIC by ligation-independent cloning (LIC) as described previously [104]. J44/J45, J46/J47, J48/J49 and J50/J51 were used for amplifying anti-sense fragment from cDNA of *N. benthamiana* for silencing *NbAGO1-1*, *NbAGO1-2*, *NbAGO4-1* and *NbAGO4-2*, respectively. pTRV2-LIC vector was digested with PstI and ligated with PCR fragments.

For BSMV mediated silencing of miR9863 members in barley, STTM-miR9863 and STTM-EV were first designed and amplified by using overlapping primer groups J52/J53/J54 and J55/J56, according to **Fig. 5A** and Yan and associates [80]. Then PCR products were cloned into pCaBS-γbLIC vector through LIC strategy as described above.

For single-cell transient overexpression of miR9863 members, the *tae-MIR9863a*, *tae-MIR9863c* or *hvu-MIR9863b* precursors with *attB* sites were cloned into pUbi-GATE vector by Gateway technology described above. Similarly, pUbi-GW-mYFP vector was used for expression of *Mla1* or *Mla10* cDNA.

### One-step site-directed mutagenesis

Point mutations for different *Mla* cDNAs or *MIRNA* precursors were introduced using one-step site-directed mutagenesis as described previously with minor modifications [105]. The entry vectors harboring wild-type *Mla* allele cDNA or 35S-pKANNIBAL vectors containing indicated wild-type *MIRNA* precursor sequences were used as templates for PCR reaction to introduce mutations.

### Real-time or semi-quantitative RT-PCR analysis

Total RNAs were extracted from plant materials using TRIzol solution, and treated with RNase-free DNase I (TaKaRa). About 2 µg of total RNA and M-MLV Reverse Transcriptase (Promega) were further used for reverse transcription. For coding genes reverse transcription, first-strand cDNA was synthesized using Oligo (dT)_18_. For small RNA reverse transcription, specifically designed stem-loop reverse transcription primers were used, and followed the procedures described by Chen and colleagues [106]. Primer J71, J72, J75 and J76 were used for miR9863a/b.2, miR9863c/b.1, phasiRNAI and phasiRNAII, respectively; primer J81 was used for *U6* (*U6* stands for *U6* spliceosomal RNA), and obtained cDNA was diluted 10 times and used for further analysis. Real-time qPCR was performed using StepOne real-time system (Applied Biosystems) and GoTaq qPCR Master Mix (Promega, A6001); for the determination of three *Mla1* amplicons (**Fig. 7**), primer pairs J65/J66, J67/J68 and J69/J70 were used; Primer J73, J74, J77 and J78 were respectively used with J79 to quantify the level of miR9863a/b.2, miR9863c/b.1, phasiRNAI and phasiRNAII; Primer pair J80/J81 and J82/J83 were used for the detection of *U6* and *Actin*. Semi-quantitative RT-PCR was performed as described previously [56]. Primer pair J61/J62, J63/J64 were designed to determine the silencing efficiency of *NbAGO1* and *NbAGO4*, respectively.

### RNA gel-blot analysis

RNA gel blot analysis was performed as described previously with minor modifications [107]. Total RNAs of 10 to 15 µg were separated on a 15% polyacrylamide denaturing gel by electrophoresis and cross-linking was performed as described previously [108]. The complementary sequences corresponding to miRNAs were used as probes after labeled with [γ-^32^P]ATP using T4 polynucleotide kinase (New England Biolabs). Probe J84, J85, J86 and J88 were used to detect the mature miR9863a, miR9863b.1, miR9863b.2 and miR156a signal, respectively; probe J87 was used for *Mla1* derived phasiRNAs in **S8 Figure** according to Chen and colleagues [44].

### *Agrobacterium*-mediated transient gene expression and protein analysis

*Agrobacterium*-mediated transient expression in *N. benthamiana* was performed as described previously [56], [109]. For single expression, *Agrobacterium* suspensions expressing gene of interest were infiltrated into 4- to 5-week old *N. benthamiana* leaves. For co-expressions, *Agrobacterium* suspensions expressing *MIRNA* precursor were first infiltrated, and 24 hrs later, *Agrobacterium* suspensions expressing gene candidate was infiltrated into the same position. Samples were collected from the infiltrated sites at 36 hrs post infiltration. Total soluble proteins were extracted using 180 µl of 2× Laemmli buffer [110] from 60 mg leaf samples, and detected by immunoblotting as described previously [56]. Rat anti-HA antibody (1∶5000; Roche, 11867423001) and anti-rat IgG conjugated with horseradish peroxidase (HRP) (1∶10000; Sigma, A5795) were used for HA-tagged proteins detection. The levels of β-actin were determined using an anti-β-actin antibody (1∶1000; CWBIO, CW0264) coupled with anti-mouse IgG conjugated with HRP (1∶75000; Sigma, A9044).

### Single-cell transient gene expression assay

Single-cell transient gene expression assay using biolistic delivery of plasmid DNA into barley epidermal cells was performed as previously described [77]. For *MIRNAs* overexpression, the β-glucuronidase (*GUS*) reporter gene was mixed with respective plasmids (molar ratio 1∶1) before coating of gold particles. Barley leaf epidermal cells were transformed with the biolistic particle delivery system (Bio-Rad, Model PDS-1000/He), and incubated 4 hrs before inoculation of *Bgh* spores. To identify transformed cells, bombarded leaves were fixed in solution at 48 hrs after *Bgh* infection and further stained for GUS activity. For *MIRNAs* and *Mla* co-expression, the GUS reporter and *MIR9863b* plasmids were co-coated with *Mla1-mYFP* or *Mla10-mYFP* plasmids (molar ratio 1∶1∶1).

### Trypan blue staining

Trypan blue staining was described previously [56]. Briefly, *N. benthamiana* leaves were boiled for 5 min in staining solution, and were then de-stained in 2.5 g ml^-1^ chloral hydrate in distilled water for at least 3 days.

### TRV and BSMV virus-induced gene silencing assay

TRV-based virus induced gene silencing assay was performed as described [56], [111]. Briefly, pTRV1 and pTRV2-derived constructs (pTRV2: EV, pTRV2: *NbPDSas*, pTRV2: *NbAGO1-1as*, pTRV2: *NbAGO1-2as*, pTRV2: *NbAGO4-1as*, pTRV2: *NbAGO4-2as*) were transformed into *A. tumefaciens* strain GV3101. Cell suspensions containing pTRV1 and pTRV2-derived constructs were collected and mixed at 1∶1 ratio and infiltrated into the third to fifth leaf of three-week-old *N. benthamiana* plants. Three weeks after infiltration, the upper newly expanded leaves were selected for further analysis.

For BSMV-mediated STTM-VIGS assay of miR9863 members, constructs of pCaBS-α, pCaBS-β, and pCaBS-γbLIC derivatives (pCaBS-γSTTM-EV and pCaBS-γSTTM-miR9863) were transformed into the *A. tumefaciens* strain EHA105, respectively. The *Agrobacterium* suspensions of OD_600_ = 0.8 were mixed at 1∶1∶1 ratio and infiltrated in *N. benthamiana* leaves. The *N. benthamiana* sap was extracted from leaves with BSMV symptom at about 12 days post infiltration, and inoculated to the first two emerging leaves of barley leaves. About 15 days later, the newly grown upper barley leaves with virus symptom were collected for further analysis.

### Accession numbers

Sequence data for genes used in this article can be found under GenBank accession numbers AY009939 (*HvuMla1*), GU245938 (*HvuMla2*), GU245939 (*HvuMla3*), AJ302292 (*HvuMla6*), AY266444 (*HvuMla7*), GU245940 (*HvuMla8*), GU245941 (*HvuMla9*), AY266445 (*HvuMla10*), AY196347 (*HvuMla12*), AF523683 (*HvuMla13-1*), GU245942 (*HvuMla16-1*), GU245943 (*HvuMla18-1*), GU245944 (*HvuMla18-2*), GU245945 (*HvuMla19-1*), GU245946 (*HvuMla22*), GU245947 (*HvuMla23*), GU245948 (*HvuMla25-1*), GU245949 (*HvuMla27-1*), GU245950 (*HvuMla27-2*), GU245951 (*HvuMla28*), GU245952 (*HvuMla30*), GU245953 (*HvuMla31-1*), GU245954 (*HvuMla32*), GU245955 (*HvuMla34*), GU245956 (*HvuMla35*), GU245957 (*HvuMla36-1*), GU245958 (*HvuMla37-1*), GU245959 (*HvuMla38-1*), GU245960 (*HvuMla39-1*), KJ619975 (*hvu-MIR9863a*), AK364228 (*hvu-MIR9863b*), CK193889 (*tae-MIR9863a*, *tae-MIR9863b*, *tae-MIR9863a/b* cluster), DR736484 (*tae-MIR9863c*), DQ321488 (*NbAGO1-1*), DQ321489 (*NbAGO1-2*), DQ321490 (*NbAGO4-1*), DQ321491 (*NbAGO4-2*), ACZ65507 (*HchMla1*), ADX06722 (*TmoMla1*), AGP75918 (*TmoSr35*), KF031291 (*AetSr33*), ACG63536 (*TduLr10*), AAG42168 (*TaeYr10*), AAY21626 (*TaePm3a*), AAQ96158 (*TaePm3b*), ABB78077 (*TaePm3c*) and AAY21627 (*TaePm3d*).

## Supporting Information

S1 Figure Sequence alignment of the miR9863 target site and RNBS-D motif of selected R proteins from barley, wheat and related species. Hch, *Hordeum chilense*; Tmo, *Triticum monococcum*; Tdu, *Triticum durum*; Aet, *Ageilops tauschii*.
(TIF)

S2 Figure *MIR9863* precursor diagrams and expression upon Agro-infiltration in *N. benthamiana.* (A) Diagram of the secondary structure of indicated *MIR9863* precursors. The miR9863 and miR9863* region is marked by red and blue, respectively; red and orange line below indicate the mature miRNA generated by the precursor. (B) The expression of various *MIR9863* precursors by Agro-infiltration in *N. benthamiana*. The indicated *MIRNA* precursor was transiently expressed in *N. benthamiana*, and the level of mature miRNAs was determined by RNA gel blot. Mixed probes were used for quantifying both miR9863b.1 and miR9863b.2, and miR156 level is shown as a loading control.
(TIF)

S3 Figure RNA gel-blot analysis for the expression of miR9863 family in different plant species. Total RNAs were obtained from different plants and electrophoresed in 15% polyacrylamide gel. Signals were derived from cross-hybridization with a mixture of probe for miR9863a/c/b.1/b.2, and miR156 and 5S rRNA are served as loading controls. Barley cultivar (Hvu): Carlsberg II, Franka, Golden promise, Pallas, Siri, Ingrid and Morex; bread wheat cultivars (Tae): Chancellor and Kenong199.
(TIF)

S4 Figure Verification of the two cleavage positions at the miR9863-binding site in *Mla1* transcript. (A) Nucleotides T to A substitution at 1266 position (T1266A), or CT to GA substitution at 1270–1271 positions (CT1270GA) were introduced into *Mla1* sequence to generate large loops between the miR9863 and its target site. Arrows indicate the cleavage position confirmed by 5′ RACE (**Fig. 1D**). (B) Wild type or mutated *Mla1* were co-expressed with *MIR9863a* in *N. benthamiana* by Agro-infiltration. MLA1 and actin protein levels were determined by immunoblotting at 36 hpai, and rubisco was used as a loading control.
(TIF)

S5 Figure *Arabidopsis MIR173* backbone was used for constructing artificial *MIR9863* precursors. The mature miR9863a, miR9863b.1 or miR9863b.2 sequences were engineered into the *Arabidopsis* miR173 precursor backbone using overlapping PCR to replace mature miR173 and miR173* sequences. miRNA and miRNA* are marked by red and blue, respectively.
(TIF)

S6 Figure Single nucleotide variation between miR9863a and miR9863b.2 determines the regulation efficiency on *Mla1.* (A) Alignment of mature miR9863a and miR9863b.2 sequences. (B) Nucleotides T to C substitution was introduced into position 9 of mature miR9863a sequence in *tae-MIR9863a*. *MIR9863a*-T9C should generate mature miR9863a-T9C sequentially equal to miR9863b.2. (C) Determination of the regulation efficiency of miR9863a and miR9863a-T9C on *Mla1*. *Tae-MIR9863a* and *tae-MIR9863a*-T9C were separately co-expressed with *Mla1* in *N. benthamiana*. MLA1 levels were quantified by immunoblotting, and rubisco was used as a loading control (panel a). The miRNA expressions were determined by RNA gel-blot using a mixture of probes for miR9863a and miR9863b.2, and the miR156 and 5S rRNA are served as loading controls (panel b).
(TIF)

S7 Figure Natural SNP variations among *Mla* alleles dictate the regulation specificity of miR9863b. *Hvu-MIR9863b* was co-expressed with indicated WT *Mla* allele or mutant variants, and experiments was done same as in **Fig. 4**. MLA and actin levels were determined by immunoblotting; Rubisco was used as a loading control.
(TIF)

S8 Figure 22-nt miR9863a triggered phasiRNAs production is required for the complete repression of MLA1_ARC domain accumulation. (A) Construct diagram for expressing MLA1_ARC-mYFP-HA fusion. Expression vector harboring cDNA sequence encoding MLA1_ARC domain was coexpressed with *MIR9863a* or *MIR9863a_21_*, and experiment was done similar to **Fig. 6E**. The horizontal line indicates a DNA oligonucleotide probe complementary to a 42-nt region downstream of the miR9863a cleavage site for detecting phasiRNAs derived from *Mla1*. (B) Determination of indicated RNA or MLA1_ARC protein level. The levels of miR9863a and phasiRNAs were detected by RNA gel-blot using probes for miR9863a and siRNAs (see supplemental **Table 2**), and miR156 and 5S rRNA are shown as loading controls (panel a). The MLA1_ARC and actin were detected by immunoblotting with anti-HA or anti-Actin antibody, and rubisco was used as the loading control (panel b).
(TIF)

S9 Figure AGO1 but not AGO4 is essential for miR9863-mediated *Mla1* regulation. (A) Sequence and position of primers for PCR amplification of fragment of alleles of *NbAGO1* or *NbAGO4*. (B) Semi-quantitative RT-PCR analysis of levels of *NbAGO1* or *NbAGO4* in TRV-silencing *N. benthamiana* plants. Empty vector (pTRV: 00) infected plants were employed as negative controls. PCR cycle numbers were indicated, and *actin* was used as a loading control. (C) Phenotypes for TRV*-*silenced *PDS*, *AGO1-1* and *AGO1-2*, or *AGO4-1* and *AGOA4-2 N. benthamiana* plants. The third to fifth leaves of *N. benthamiana* plant were infiltrated with Agrobacteria containing pTRV vectors harboring antisense fragment of *NbPDS*, *NbAGO1-1*, *NbAGO1-2*, *NbAGO4-1* or *NbAGO4-2* as described in Materials and Methods. Photobleaching is observed on upper leaves of *NbPDS*-silenced plant. pTRV: 00 treated plant is shown as a negative control. (D) miR9863-mediated regulation on *Mla1* in *AGO1*- or *AGO4*-silenced *N. benthamiana* plants. *Hvu-MIR9863b* and *tae-MIR9863a* was each co-expressed with *Mla1-3HA* in indicated TRV*-*silencing plant, and the levels of MLA1 were determined by immunoblotting at 36 hpai using actin as a loading control (panel a). The expression of miR9863b.1/b.2 or miR9863a was determined by RNA gel blot with a mixture of probes for miR9863a and miR9863b.1. miR156 and 5S rRNA were used as controls (panel b).
(TIF)

S10 Figure *Mla1* expression level is inversely correlated with the abundance of mature miR9863s and phasiRNAs during incompatible interactions. (A) Expression of *Mla1* during *Bgh* infections. A barley transgenic line expressing functional *Mla1*-HA fusion driven by its native promoter was infected with avirulence *Bgh* K1 (*AVR_a1_*), and *Mla1* transcript levels were determined by qRT-PCR using primers for *Mla1* amplicon 2 (see **Fig. 7** and supplemental **Table 2**). (B) Expression of miR9863c/b.1 or miR9863a/b.2 during *Bgh* infections. Expression levels of mature miR9863 members were detected by stem-loop qRT-PCR with specifically designed primers (see supplemental **Table 2**). (C) The accumulation of phasiRNAI and phasiRNAII during *Bgh* infections. PhasiRNAI and phasiRNAII were first reverse transcribed by specific RT primers (listed in supplemental **Table 2**), and quantified by stem-loop qRT-PCR. Data from different time points in (A) were all normalized to *actin* levels, while data in (B) and (C) were normalized to *U6*. Relative expression level in (A) to (C) at each time point was calculated by comparing to time point 0 hpi. ‘*’ and ‘**’ above the bars indicate significant differences at *p*<0.05 and *p*<0.01, respectively. All experiments were conducted at least twice with similar results.
(TIF)

S11 Figure Phylogeny analysis of full-length or partial *Mla* cDNA sequences. The 29 *Mla* full-length cDNAs (A), cDNAs encoding CC (B), NB-ARC (C), or LRR (D) domains are used for the NJ distance tree analyses. *Mla* members from group I, II and III (see **Fig. 3**) are marked by red dot, green rectangle and blue lozenge, separately; Red triangles on the right in **(C)** mark the RAR1-dependent *Mla* alleles.
(TIF)

S1 Table miR9863 family members annotated in previous studies.
(DOCX)

S2 Table Primers and probes used in this study.
(DOCX)

S3 Table Plasmids and constructs used in this study.
(DOCX)
