# Supplementary figures and images for: The miR9863 Family Regulates Distinct Mla Alleles in Barley to Attenuate NLR Receptor-Triggered Disease Resistance and Cell-Death Signaling
Source: PLoS Genet. 2014 Dec 11;10(12):e1004755. doi: 10.1371/journal.pgen.1004755 (PMC4263374; doi:10.1371/journal.pgen.1004755)

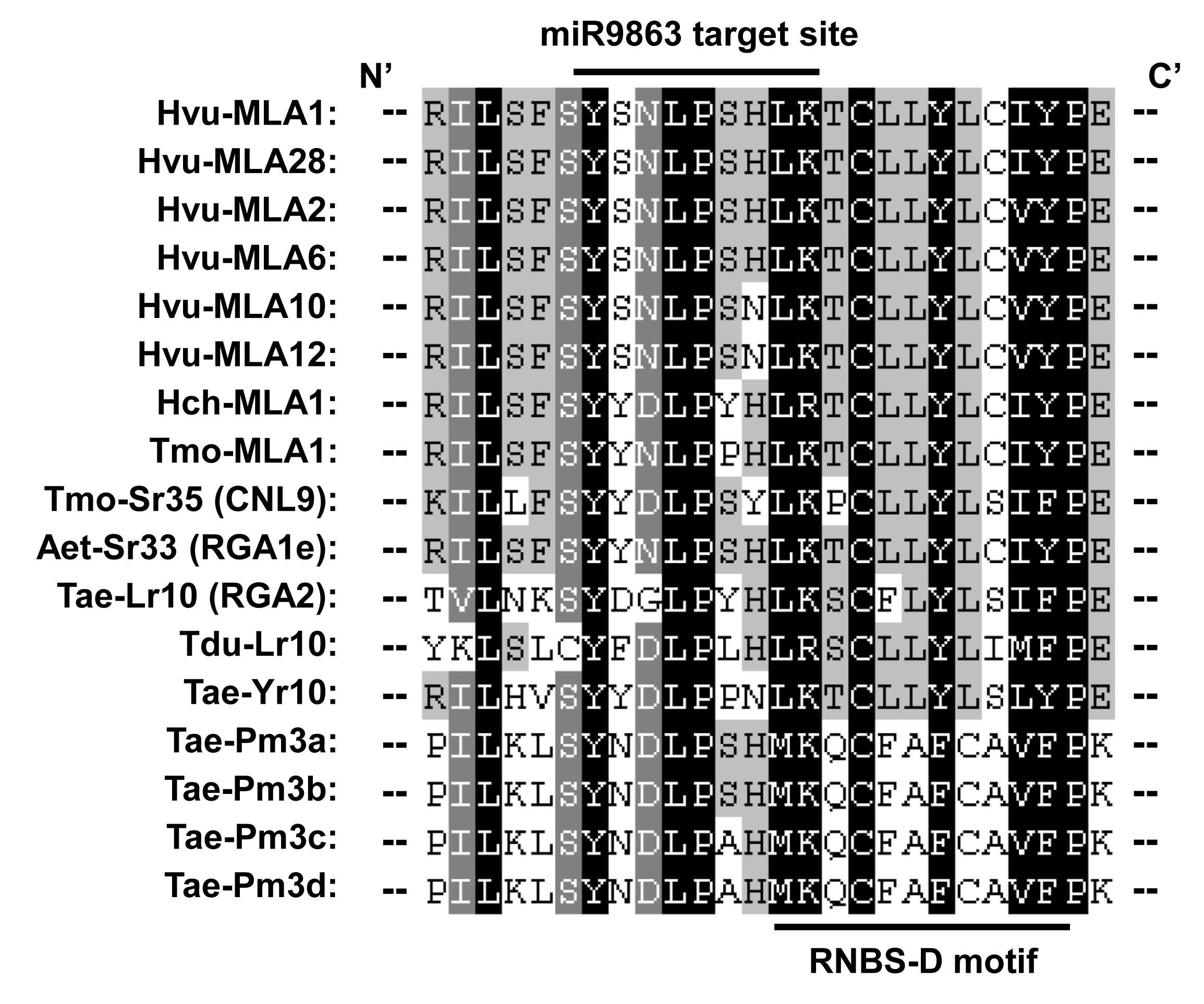

Supplement: S1 Figure — Sequence alignment of the miR9863 target site and RNBS-D motif of selected R proteins from barley, wheat and related species. Hch, Hordeum chilense; Tmo, Triticum monococcum; Tdu, Triticum durum; Aet, Ageilops tauschii. (TIF) [file pgen.1004755.s001.tif]

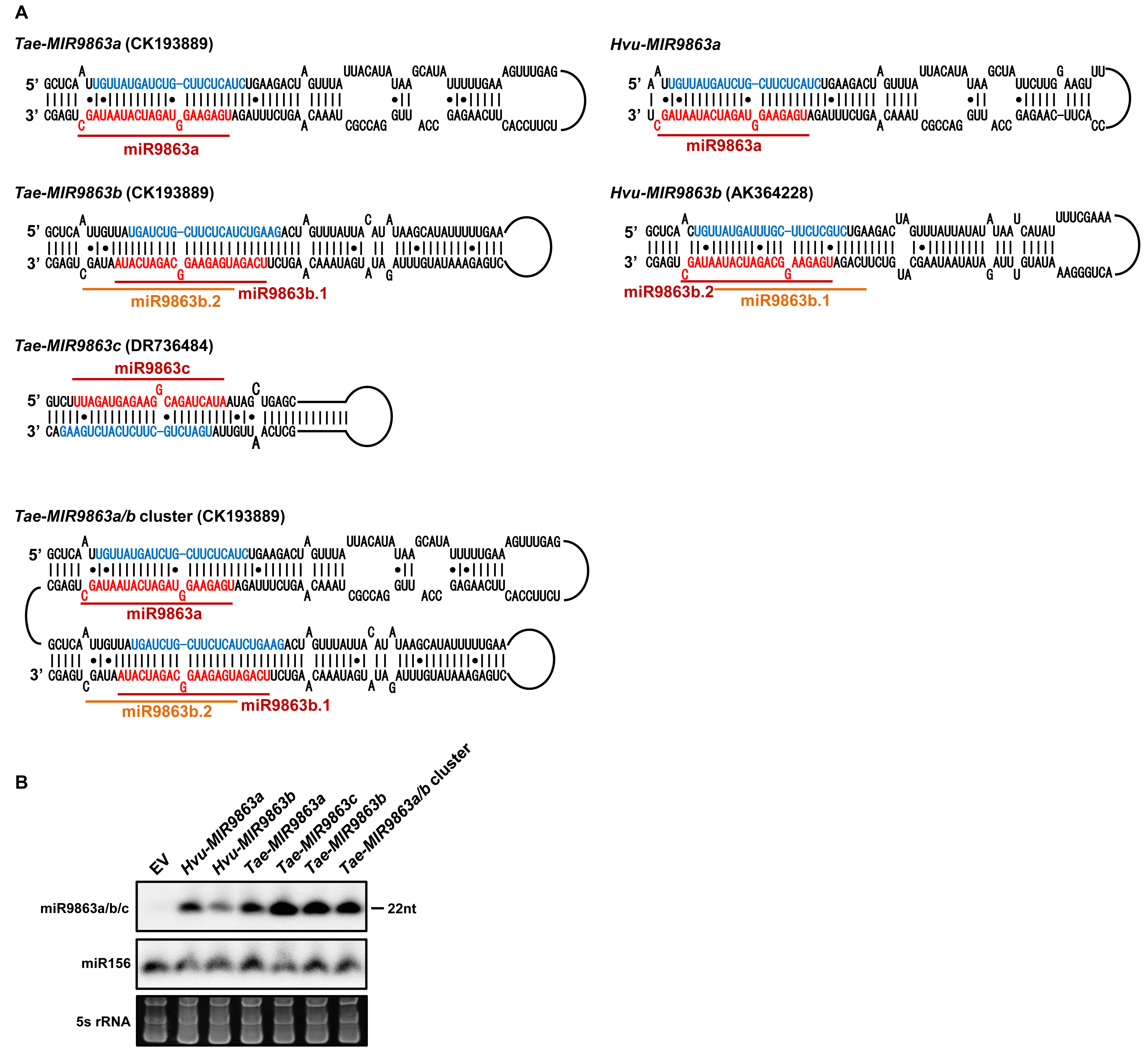

Supplement: S2 Figure — MIR9863 precursor diagrams and expression upon Agro-infiltration in N. benthamiana. (A) Diagram of the secondary structure of indicated MIR9863 precursors. The miR9863 and miR9863* region is marked by red and blue, respectively; red and orange line below indicate the mature miRNA generated by the precursor. (B) The expression of various MIR9863 precursors by Agro-infiltration in N. benthamiana. The indicated MIRNA precursor was transiently expressed in N. benthamiana, and the level of mature miRNAs was determined by RNA gel blot. Mixed probes were used for quantifying both miR9863b.1 and miR9863b.2, and miR156 level is shown as a loading control. (TIF) [file pgen.1004755.s002.tif]

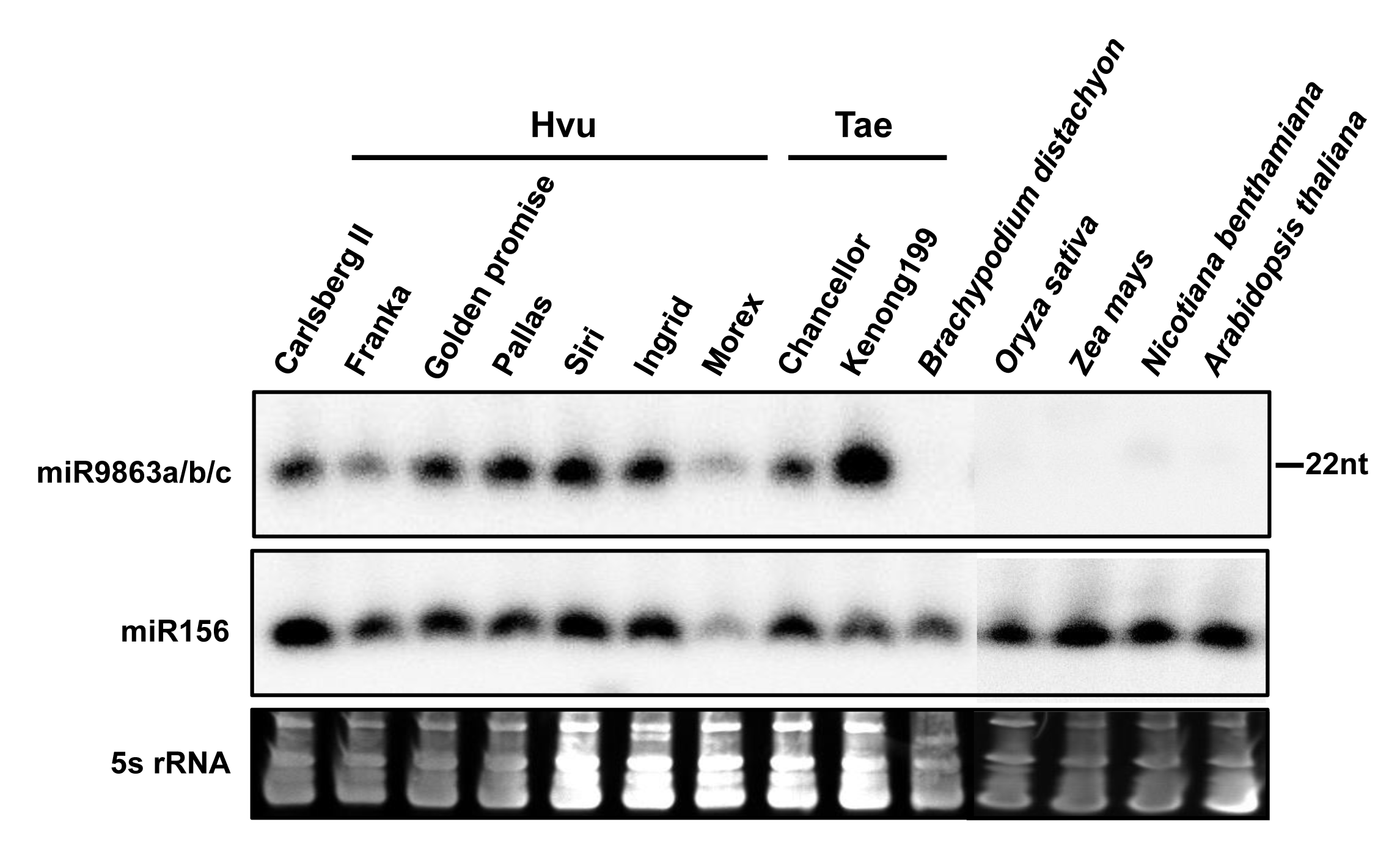

Supplement: S3 Figure — RNA gel-blot analysis for the expression of miR9863 family in different plant species. Total RNAs were obtained from different plants and electrophoresed in 15% polyacrylamide gel. Signals were derived from cross-hybridization with a mixture of probe for miR9863a/c/b.1/b.2, and miR156 and 5S rRNA are served as loading controls. Barley cultivar (Hvu): Carlsberg II, Franka, Golden promise, Pallas, Siri, Ingrid and Morex; bread wheat cultivars (Tae): Chancellor and Kenong199. (TIF) [file pgen.1004755.s003.tif]

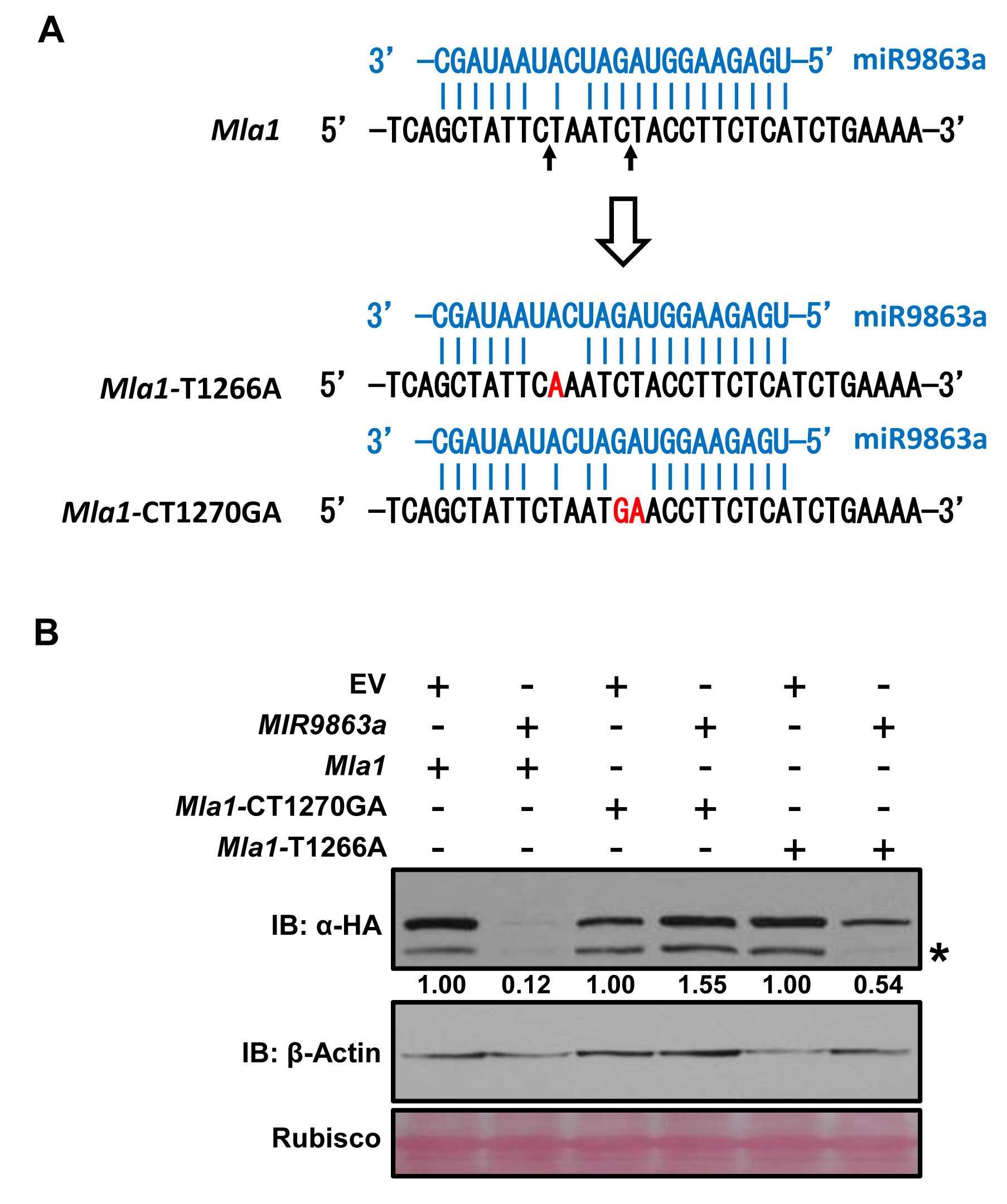

Supplement: S4 Figure — Verification of the two cleavage positions at the miR9863-binding site in Mla1 transcript. (A) Nucleotides T to A substitution at 1266 position (T1266A), or CT to GA substitution at 1270–1271 positions (CT1270GA) were introduced into Mla1 sequence to generate large loops between the miR9863 and its target site. Arrows indicate the cleavage position confirmed by 5′ RACE ( Fig. 1D ). (B) Wild type or mutated Mla1 were co-expressed with MIR9863a in N. benthamiana by Agro-infiltration. MLA1 and actin protein levels were determined by immunoblotting at 36 hpai, and rubisco was used as a loading control. (TIF) [file pgen.1004755.s004.tif]

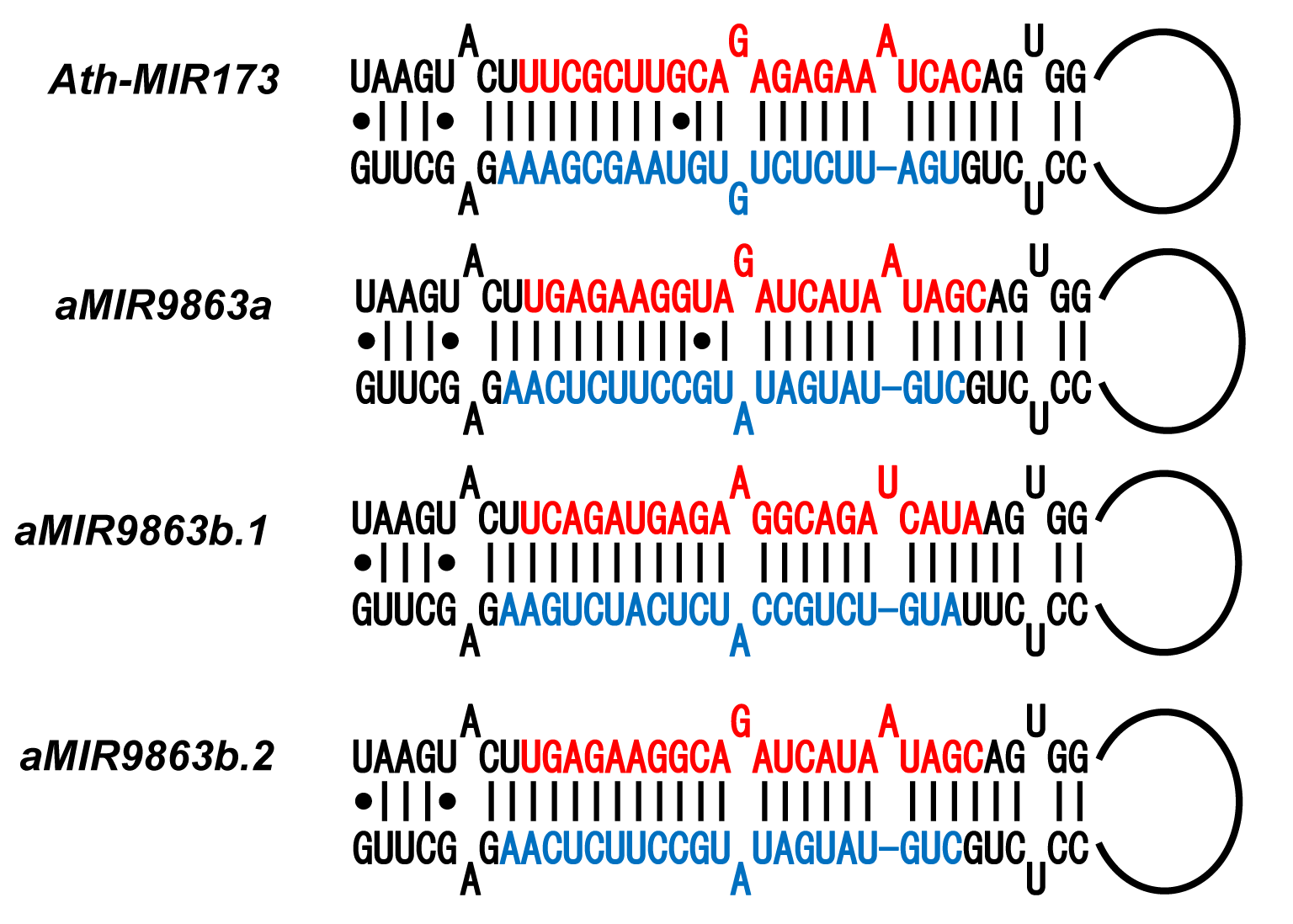

Supplement: S5 Figure — Arabidopsis MIR173 backbone was used for constructing artificial MIR9863 precursors. The mature miR9863a, miR9863b.1 or miR9863b.2 sequences were engineered into the Arabidopsis miR173 precursor backbone using overlapping PCR to replace mature miR173 and miR173* sequences. miRNA and miRNA* are marked by red and blue, respectively. (TIF) [file pgen.1004755.s005.tif]

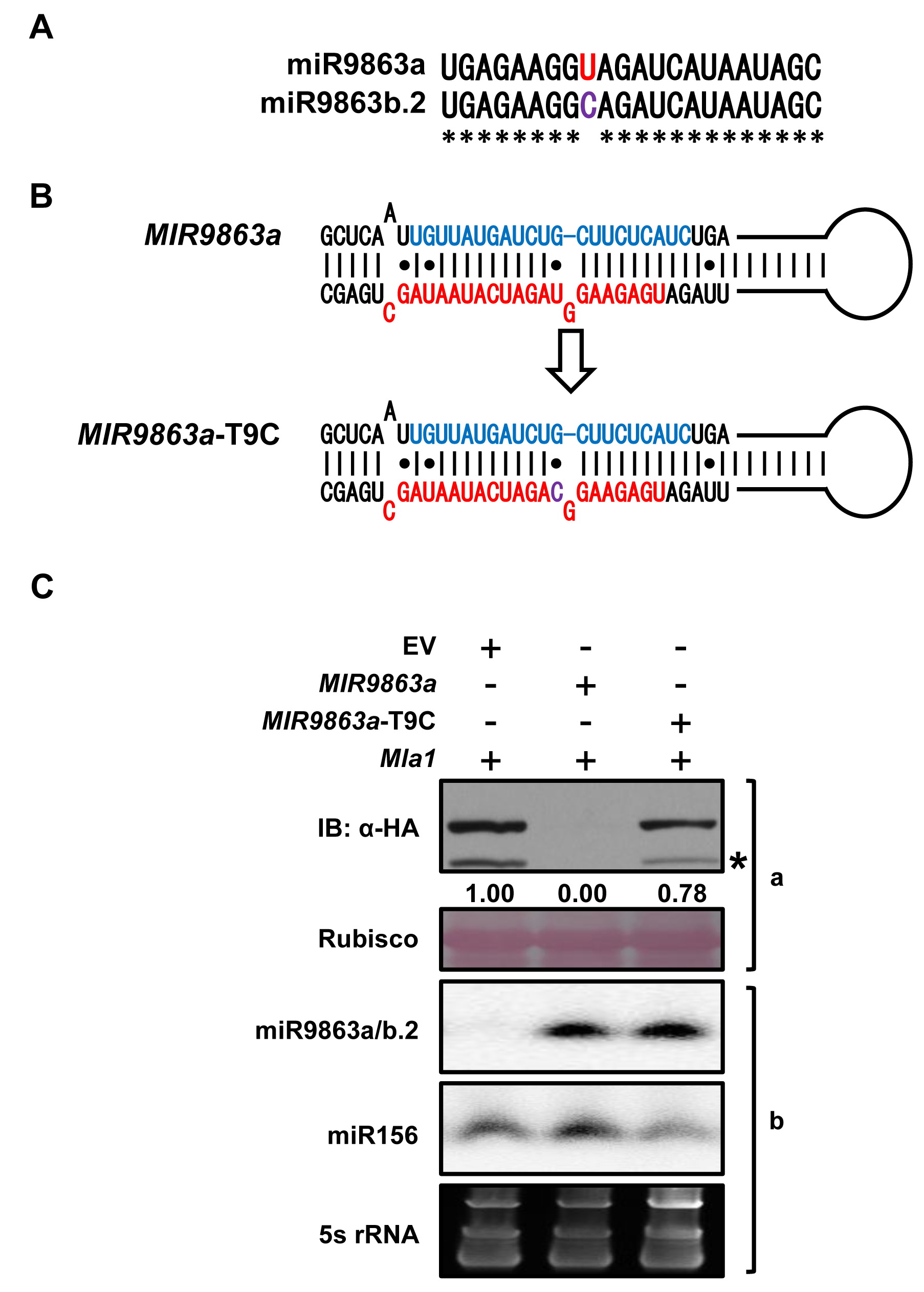

Supplement: S6 Figure — Single nucleotide variation between miR9863a and miR9863b.2 determines the regulation efficiency on Mla1. (A) Alignment of mature miR9863a and miR9863b.2 sequences. (B) Nucleotides T to C substitution was introduced into position 9 of mature miR9863a sequence in tae-MIR9863a. MIR9863a-T9C should generate mature miR9863a-T9C sequentially equal to miR9863b.2. (C) Determination of the regulation efficiency of miR9863a and miR9863a-T9C on Mla1. Tae-MIR9863a and tae-MIR9863a-T9C were separately co-expressed with Mla1 in N. benthamiana. MLA1 levels were quantified by immunoblotting, and rubisco was used as a loading control (panel a). The miRNA expressions were determined by RNA gel-blot using a mixture of probes for miR9863a and miR9863b.2, and the miR156 and 5S rRNA are served as loading controls (panel b). (TIF) [file pgen.1004755.s006.tif]

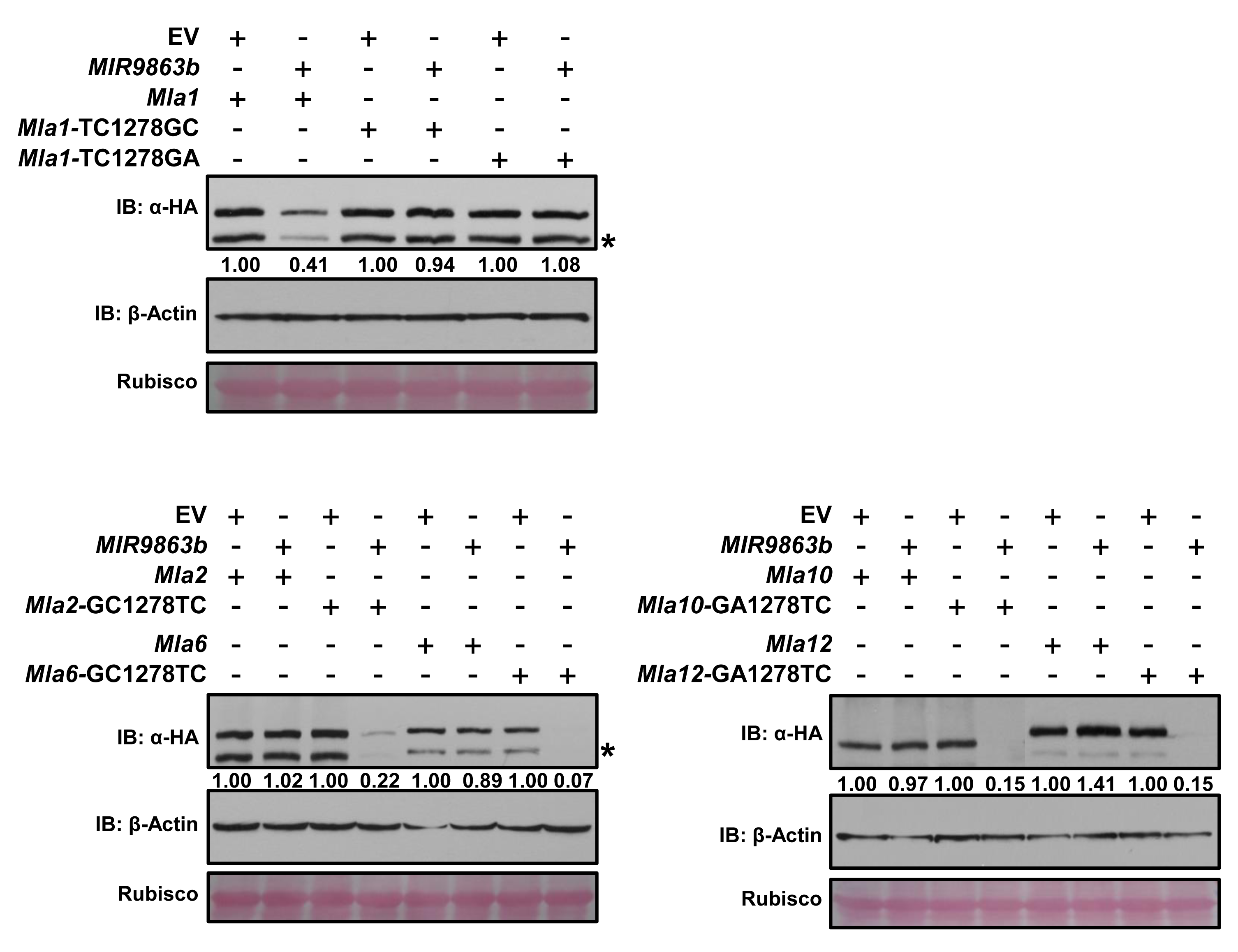

Supplement: S7 Figure — Natural SNP variations among Mla alleles dictate the regulation specificity of miR9863b. Hvu-MIR9863b was co-expressed with indicated WT Mla allele or mutant variants, and experiments was done same as in Fig. 4 . MLA and actin levels were determined by immunoblotting; Rubisco was used as a loading control. (TIF) [file pgen.1004755.s007.tif]

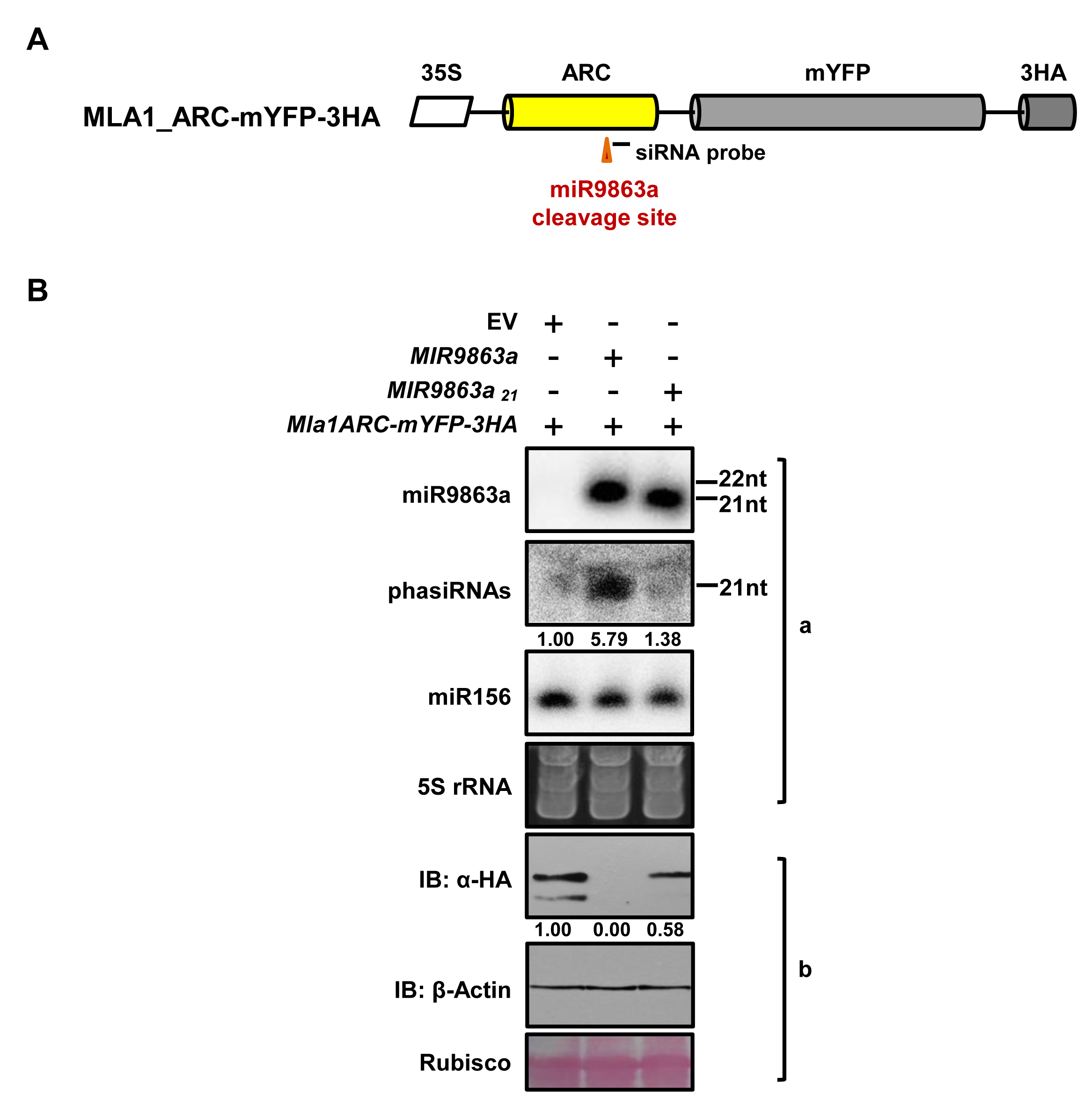

Supplement: S8 Figure — 22-nt miR9863a triggered phasiRNAs production is required for the complete repression of MLA1_ARC domain accumulation. (A) Construct diagram for expressing MLA1_ARC-mYFP-HA fusion. Expression vector harboring cDNA sequence encoding MLA1_ARC domain was coexpressed with MIR9863a or MIR9863a21, and experiment was done similar to Fig. 6E . The horizontal line indicates a DNA oligonucleotide probe complementary to a 42-nt region downstream of the miR9863a cleavage site for detecting phasiRNAs derived from Mla1. (B) Determination of indicated RNA or MLA1_ARC protein level. The levels of miR9863a and phasiRNAs were detected by RNA gel-blot using probes for miR9863a and siRNAs (see supplemental Table 2), and miR156 and 5S rRNA are shown as loading controls (panel a). The MLA1_ARC and actin were detected by immunoblotting with anti-HA or anti-Actin antibody, and rubisco was used as the loading control (panel b). (TIF) [file pgen.1004755.s008.tif]

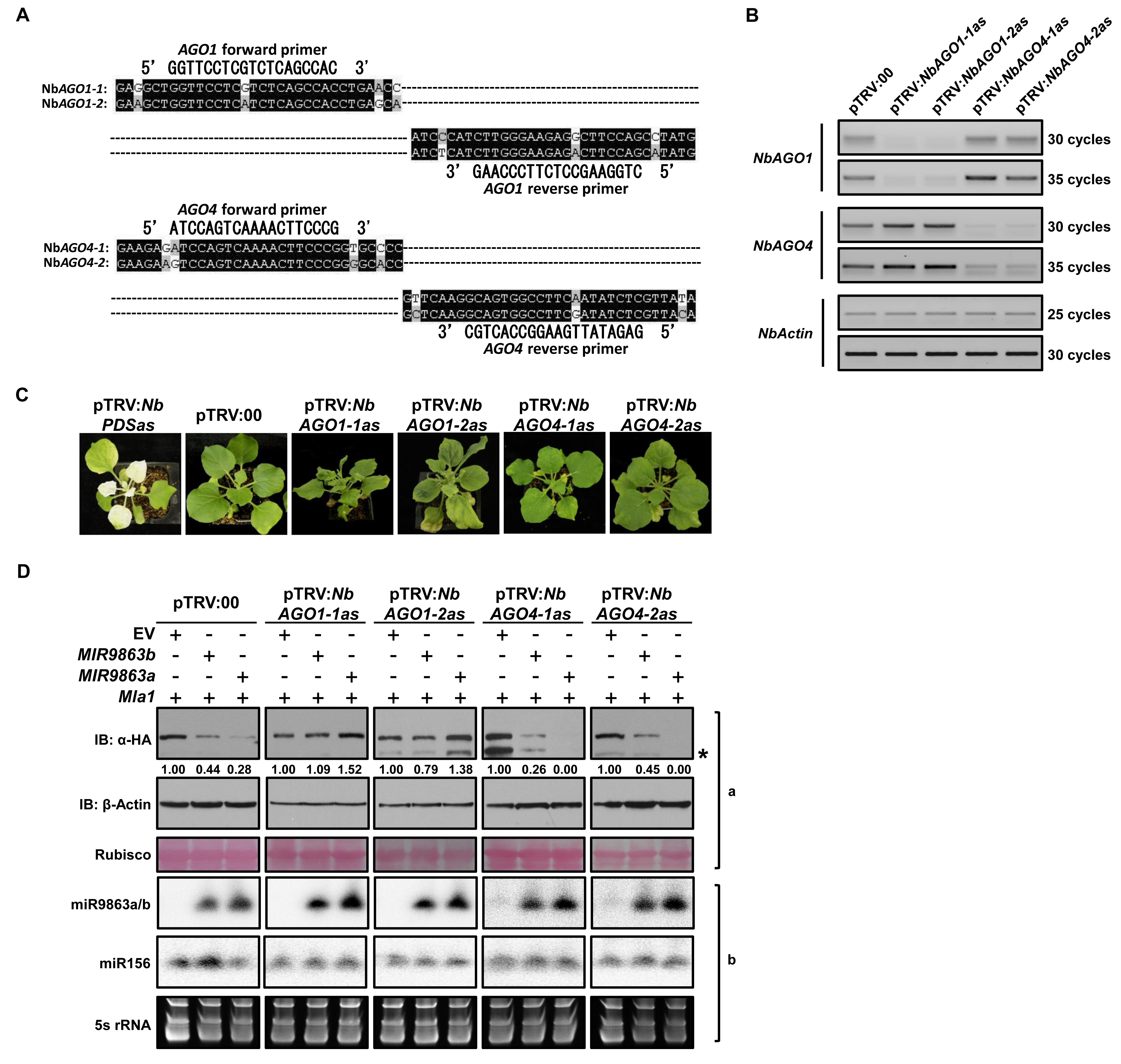

Supplement: S9 Figure — AGO1 but not AGO4 is essential for miR9863-mediated Mla1 regulation. (A) Sequence and position of primers for PCR amplification of fragment of alleles of NbAGO1 or NbAGO4. (B) Semi-quantitative RT-PCR analysis of levels of NbAGO1 or NbAGO4 in TRV-silencing N. benthamiana plants. Empty vector (pTRV: 00) infected plants were employed as negative controls. PCR cycle numbers were indicated, and actin was used as a loading control. (C) Phenotypes for TRV-silenced PDS, AGO1-1 and AGO1-2, or AGO4-1 and AGOA4-2 N. benthamiana plants. The third to fifth leaves of N. benthamiana plant were infiltrated with Agrobacteria containing pTRV vectors harboring antisense fragment of NbPDS, NbAGO1-1, NbAGO1-2, NbAGO4-1 or NbAGO4-2 as described in Materials and Methods. Photobleaching is observed on upper leaves of NbPDS-silenced plant. pTRV: 00 treated plant is shown as a negative control. (D) miR9863-mediated regulation on Mla1 in AGO1- or AGO4-silenced N. benthamiana plants. Hvu-MIR9863b and tae-MIR9863a was each co-expressed with Mla1-3HA in indicated TRV-silencing plant, and the levels of MLA1 were determined by immunoblotting at 36 hpai using actin as a loading control (panel a). The expression of miR9863b.1/b.2 or miR9863a was determined by RNA gel blot with a mixture of probes for miR9863a and miR9863b.1. miR156 and 5S rRNA were used as controls (panel b). (TIF) [file pgen.1004755.s009.tif]

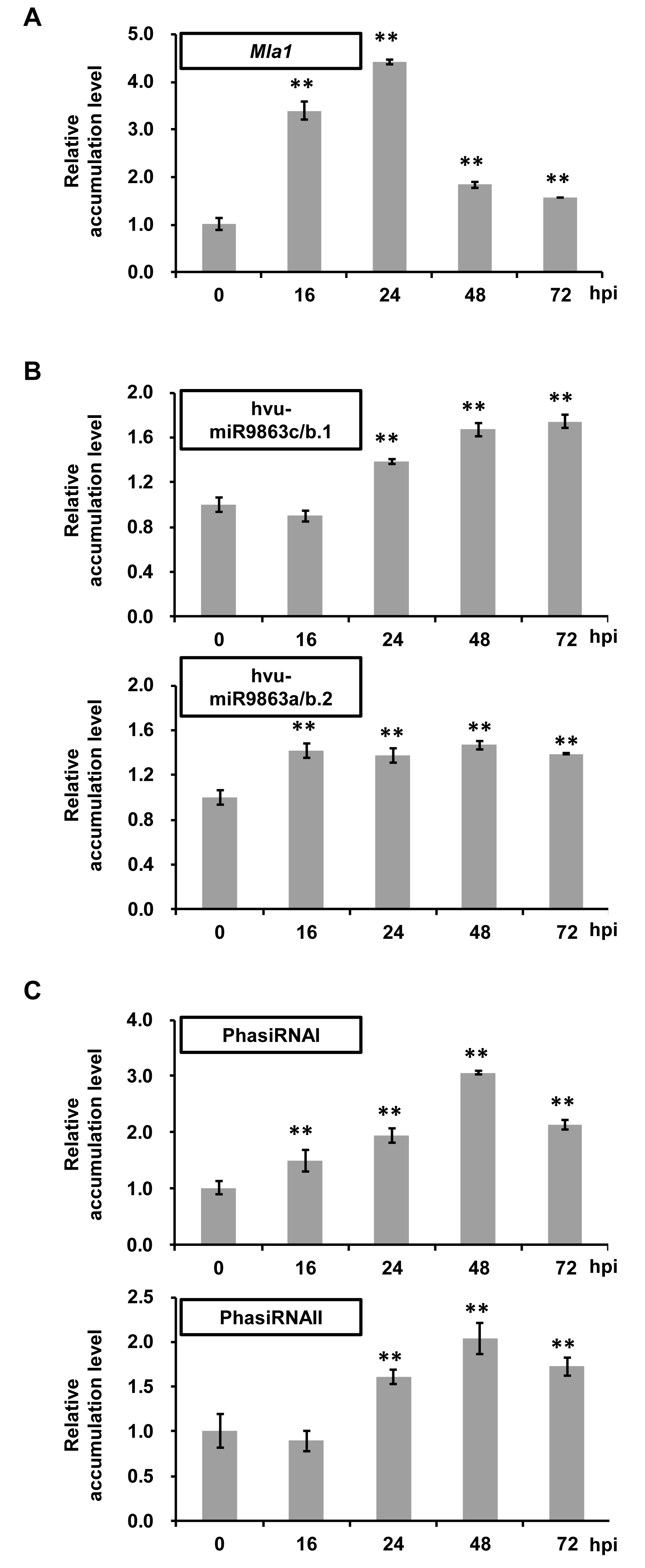

Supplement: S10 Figure — Mla1 expression level is inversely correlated with the abundance of mature miR9863s and phasiRNAs during incompatible interactions. (A) Expression of Mla1 during Bgh infections. A barley transgenic line expressing functional Mla1-HA fusion driven by its native promoter was infected with avirulence Bgh K1 (AVRa1), and Mla1 transcript levels were determined by qRT-PCR using primers for Mla1 amplicon 2 (see Fig. 7 and supplemental Table 2). (B) Expression of miR9863c/b.1 or miR9863a/b.2 during Bgh infections. Expression levels of mature miR9863 members were detected by stem-loop qRT-PCR with specifically designed primers (see supplemental Table 2). (C) The accumulation of phasiRNAI and phasiRNAII during Bgh infections. PhasiRNAI and phasiRNAII were first reverse transcribed by specific RT primers (listed in supplemental Table 2), and quantified by stem-loop qRT-PCR. Data from different time points in (A) were all normalized to actin levels, while data in (B) and (C) were normalized to U6. Relative expression level in (A) to (C) at each time point was calculated by comparing to time point 0 hpi. ‘*’ and ‘**’ above the bars indicate significant differences at p<0.05 and p<0.01, respectively. All experiments were conducted at least twice with similar results. (TIF) [file pgen.1004755.s010.tif]

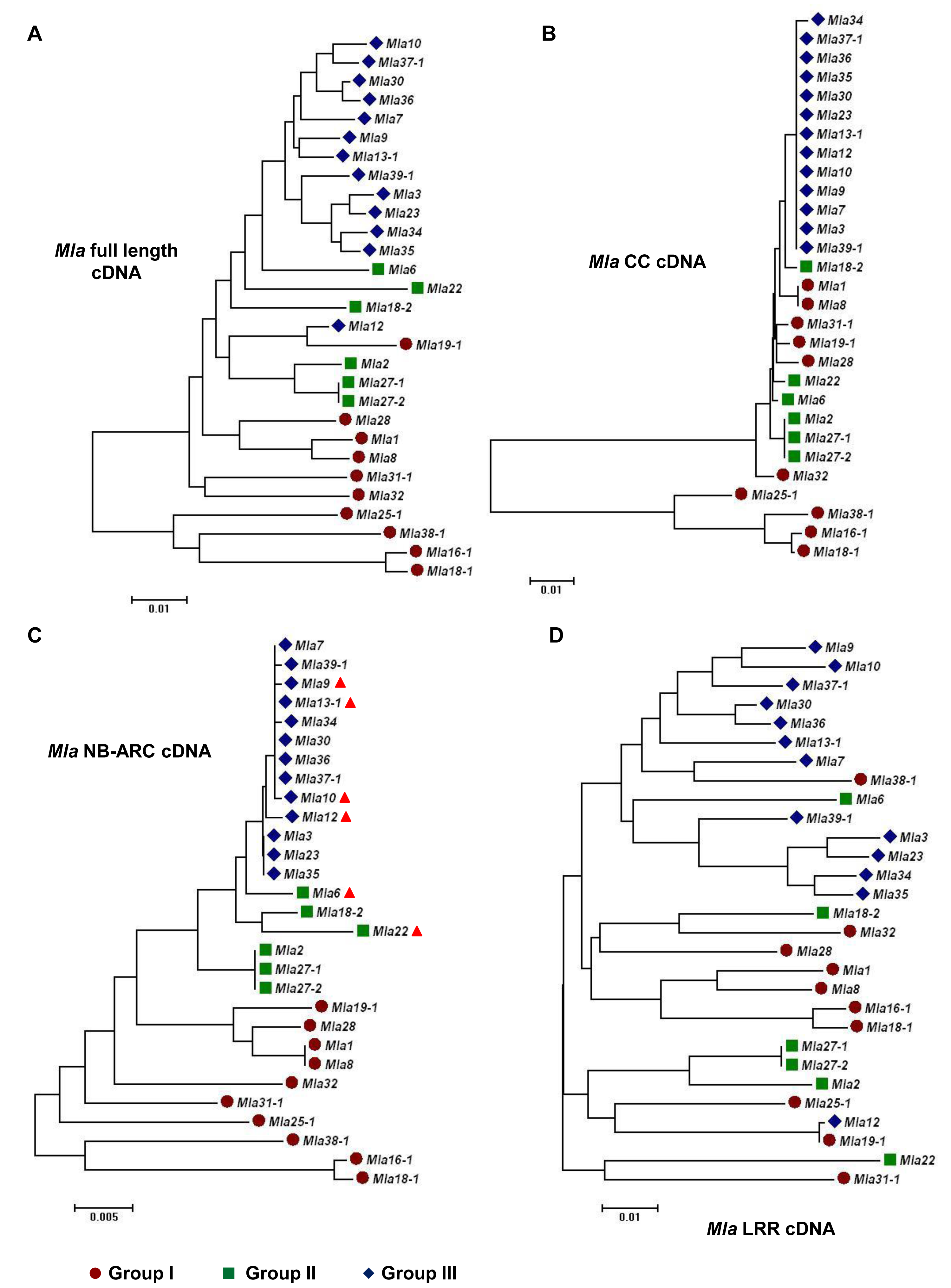

Supplement: S11 Figure — Phylogeny analysis of full-length or partial Mla cDNA sequences. The 29 Mla full-length cDNAs (A), cDNAs encoding CC (B), NB-ARC (C), or LRR (D) domains are used for the NJ distance tree analyses. Mla members from group I, II and III (see Fig. 3 ) are marked by red dot, green rectangle and blue lozenge, separately; Red triangles on the right in (C) mark the RAR1-dependent Mla alleles. (TIF) [file pgen.1004755.s011.tif]
