# Supplementary material for: The miR9863 Family Regulates Distinct Mla Alleles in Barley to Attenuate NLR Receptor-Triggered Disease Resistance and Cell-Death Signaling
Source: PLoS Genet. 2014 Dec 11;10(12):e1004755. doi: 10.1371/journal.pgen.1004755 (PMC4263374; doi:10.1371/journal.pgen.1004755)
Supplement: S1 Table — miR9863 family members annotated in previous studies. (DOCX) [file pgen.1004755.s012.docx]

**Table S1 miR9863 family members annotated in previous studies**

| **miRNAs name in other papers** | **Sequence** | **Abundance**  **(TPM)** | **Alignment to EST/genome contigs** | **Reference** | **miRNAs name in this paper** |
| --- | --- | --- | --- | --- | --- |
| tae-miR2009a | UGAGAAGGUAGAUCAUAAUAGC | 353 | CK193889, CK206502 | [[1](#_ENREF_1)] | tae-miR9863a |
| tae-miR2009b | UUAGAUGAGAAGGCAGAUCAUA | 86 | DR736484 |  | tae-miR9863c |
| tae-miR2009c | UCAGAUGAGAAGGCAGAUCAUA | 82 | CK193889 |  | tae-miR9863b.1 |
| Not annotated | UGAGAAGGCAGAUCAUAAUAGC | 52 | CK193889 |  | tae-miR9863b.2 |
| hvu-miR2009a | UGAGAAGGUAGAUCAUAAUAGC | 334 |  | [[2](#_ENREF_2)] | hvu-miR9863a |
| hvu-miR2009b | UUAGAUGAGAAGGCAGAUCAUA | 20 |  |  | hvu-miR9863c |
| hvu-miR2009c | UCAGAUGAGAAGGCAGAUCAUA | 370 | morex contig 44305, barke contig 2781255, bowman contig 15286 | [[2-4](#_ENREF_2)] | hvu-miR9863b.1 |
| hvu-miR2009d | UGAGAAGGCAGAUCAUAAUAGC | 10206 | morex contig 44305, barke contig 2781255, bowman contig 15286 |  | hvu-miR9863b.2 |
| hvu-miR2009e(*) | UUCAGAUGAGAAGGCAGAUCA | 84 | morex contig 44305, barke contig 2781255, bowman contig 15286 |  | Not annotated |

*We consider this formally annotated hvu-miR2009e of 21-nt as a by-product generated from *hvu-MIR9863b* precursor, rather than a member of the miR9863 family, thus not annotated and further analyzed in the present study.

**References**

1. Wei B, Cai T, Zhang R, Li A, Huo N, et al. (2009) Novel microRNAs uncovered by deep sequencing of small RNA transcriptomes in bread wheat (Triticum aestivum L.) and Brachypodium distachyon (L.) Beauv. Funct Int Genomics 9: 499 - 511.

2. Schreiber A, Shi B-J, Huang C-Y, Langridge P, Baumann U (2011) Discovery of barley miRNAs through deep sequencing of short reads. BMC Genomics 12: 129-149.

3. Mahalingam G, Meyers BC (2010) Computational methods for comparative analysis of plant small RNAs. Methods Mol Biol 592: 163-181.

4. Mayer KF, Waugh R, Brown JW, Schulman A, Langridge P, et al. (2012) A physical, genetic and functional sequence assembly of the barley genome. Nature 491: 711-716.
