# Supplementary material for: The miR9863 Family Regulates Distinct Mla Alleles in Barley to Attenuate NLR Receptor-Triggered Disease Resistance and Cell-Death Signaling
Source: PLoS Genet. 2014 Dec 11;10(12):e1004755. doi: 10.1371/journal.pgen.1004755 (PMC4263374; doi:10.1371/journal.pgen.1004755)
Supplement: S2 Table — Primers and probes used in this study. (DOCX) [file pgen.1004755.s013.docx]

**Table S2. Primers and probes used in this study**

| **Name** | **Sequence** | **Description** |
| --- | --- | --- |
| J01 | 5'CATGGCTACATGCTGACAGCCTA3' | 5' RACE Outer Primer |
| J02 | 5'CGCGGATCCACAGCCTACTGATGATCAGTCGATG3' | 5' RACE Inner Primer |
| J03 | 5'TGAAGGGACAGACGGCGAC3' | *Mla1* gene specific outer primer |
| J04 | 5'TGCTATTCCCACTGCCATCC3' | *Mla1* gene specific inner primer |
| J05 | 5'TTGAAGGAAATGAAAGGGAT3' | Cloning of tae-MIR9863a/b cluster; forward primer |
| J06 | 5'GTTTGGAAGAAAACAAAATG3' | Cloning of tae-MIR-9863a/b cluster; reverse primer |
| J07 | 5'CCCAGTCGTGCCGGTCTT3' | Cloning of tae-MIR9863c; forward primer |
| J08 | 5'GCTTGTGATCTCTCTCTCTCTC3' | Cloning of tae-MIR9863c; reverse primer |
| J09 | 5'GGGGACAAGTTTGTACAAAAAAGCAGGCTTCGGTACCTTGAAGGAAATGAAAGGGAT3' | Cloning of attB-KpnI-tae-MIR9863a, attB-KpnI-tae-MIR9863a/b; forward primer |
| J10 | 5'GGGGACCACTTTGTACAAGAAAGCTGGGTCAAGCTTTTCTGCACGGGATCTGCTT3' | Cloning of attB-HindIII-tae-MIR9863a; reverse primer |
| J11 | 5'GGGGACAAGTTTGTACAAAAAAGCAGGCTTCGGTACCCCCAGTCGTGCCGGTCTT3' | Cloning of attB-KpnI-tae-MIR9863c; forward primer |
| J12 | 5'GGGGACCACTTTGTACAAGAAAGCTGGGTCAAGCTTGCTTGTGATCTCTCTCTCTCTC3' | Cloning of attB-HindIII-tae-MIR9863c; reverse primer |
| J13 | 5'GGGGACAAGTTTGTACAAAAAAGCAGGCTTCGGTACCGCTGAGCGAAATGAAAGTG3' | Cloning of attB-KpnI-tae-MIR9863b; forward primer |
| J14 | 5'GGGGACCACTTTGTACAAGAAAGCTGGGTCAAGCTTGTTTGGAAGAAAACAAAATG3' | Cloning of attB-HindIII-tae-MIR9863b, attB-HindIII-tae-MIR9863a/b cluster; reverse primer |
| J15 | 5'CTCAATTTTATGTGTCGTGGG3' | Cloning of hvu-MIR9863b; forward primer |
| J16 | 5' CTGTGGGGTTACAAAAGACG 3' | Cloning of hvu-MIR9863b; reverse primer |
| J17 | 5'GGGGACAAGTTTGTACAAAAAAGCAGGCTTCGGTACCCTCAATTTTATGTGTCGTGGG3' | Cloning of attB-KpnI-hvu-MIR9863b; forward primer |
| J18 | 5'GGGGACCACTTTGTACAAGAAAGCTGGGTCAAGCTTCTGTGGGGTTACAAAAGACG3' | Cloning of attB-HindIII-hvu-MIR9863b; reverse primer |
| J19 | 5'CACCGGTACCGAAAGGGATATTGTTGCTCAA3' | Cloning of KpnI-hvu-MIR9863a; forward primer |
| J20 | 5'TAAGAAGCTTCAATATCACTTTCATTTCGCTC3' | Cloning of HindIII-hvu-MIR9863a; reverse primer |
| J21 | 5'TTCTAATCTACCTTCTCATCTGAAAACTTGTC3' | Generation of 'GC' to 'TC', or 'GA' to 'TC' mutation in *Mla2*, *Mla6*, *Mla10* or *Mla12* at 1278 nt position; forward primer |
| J22 | 5'GACAAGTTTTCAGATGAGAAGGTAGATTAGAA3' | Generation of 'GC' to 'TC', or 'GA' to 'TC' mutation in *Mla2*, *Mla6*, *Mla10* or *Mla12* at 1278 nt position; reverse primer |
| J23 | 5'TTCTAATCTACCTTCgCATCTGAAAACTTGTC3' | Generation of 'TC' to 'GC' mutation in *Mla1* at 1278 nt position; forward primer |
| J24 | 5'GACAAGTTTTCAGATGcGAAGGTAGATTAGAA3' | Generation of 'TC' to 'GC' mutation in *Mla1* at 1278 nt position; reverse primer |
| J25 | 5'TTCTAATCTACCTTCgaATCTGAAAACTTGTC3' | Generation of 'TC' to 'GA' mutation in *Mla1* at 1278 nt position; forward primer |
| J26 | 5'GACAAGTTTTCAGATtcGAAGGTAGATTAGAA3' | Generation of 'TC' to 'GA' mutation in *Mla1* at 1278 nt position; reverse primer |
| J27 | 5'TTTCAGCTATTCTAATgaACCTTCTCATCTG3' | Generation of 'CT' to 'GA' mutation in *Mla1* at 1270 nt position; forward primer |
| J28 | 5'CAGATGAGAAGGTtcATTAGAATAGCTGAAA3' | Generation of 'CT' to 'GA' mutation in *Mla1* at 1270 nt position; reverse primer |
| J29 | 5'CTCTTTCAGCTATTCaAATCTACCTTCTCA3' | Generation of 'T' to 'A' mutation in *Mla1* at 1266 nt position; forward primer |
| J30 | 5'TGAGAAGGTAGATTtGAATAGCTGAAAGAG3' | Generation of 'T' to 'A' mutation in *Mla1* at 1266 nt position; reverse primer |
| J31 | 5'CTTCGGTACCTGGTGATTAAGTACTTCAGATGAGAAGGCAGATCATAAGTGGTCAAAA3' | Construction of *aMIR9863b.1* |
| J32 | 5'CAGACATAAGAGGAAAGAGACTTTAAGAAAACTACAACTTTTTTGACCACTTATGATC3' | Construction of *aMIR9863b.1* |
| J33 | 5'CTTCAAGCTTAAGGGAGCAAGCTCTTCAGATGAGATGGCAGACATAAGAGGAAAGA3' | Construction of *aMIR9863b.1* |
| J34 | 5'CTTCGGTACCTGGTGATTAAGTACTTGAGAAGGCAGATCATAATAGCAGTGGTCAAAA3' | Construction of *aMIR9863b.2* |
| J35 | 5'CATACAGCAGAGGAAAGAGACTTTAAGAAAACTACAACTTTTTTGACCACTGCTATTA3' | Construction of *aMIR9863b.2* or *aMIR9863a* |
| J36 | 5'CTTCAAGCTTAAGGGAGCAAGCTCTTGAGAAGGCATATCATACAGCAGAGGAAAGA3' | Construction of *aMIR9863b.2* or *aMIR9863a* |
| J37 | 5'CTTCGGTACCTGGTGATTAAGTACTTGAGAAGGTAGATCATAATAGCAGTGGTCAAAA3' | Construction of *aMIR9863a* |
| J38 | 5'GATGAGAAGGCAGATCATAATAGCTGAGCGAAATG3' | Generation of 'T' to 'C' mutation at position 9 of mature miR9863a; forward primer |
| J39 | 5'TTATGATCTGCCTTCTCATCTAAAGACTTGTTTAG3' | Generation of 'T' to 'C' mutation at position 9 of mature miR9863a; reverse primer |
| J40 | 5'TATGATTTGCCTTCTCGTCTGAAGACTAGTTTATT3' | Generation of MIR9863b(21nt) to express 21-nt shortened miR9863b; forward primer |
| J41 | 5'GACGAGAAGGCAAATCATAACAGTTGAGCAACAAT3' | Generation of MIR9863b(21nt) to express 21-nt shortened miR9863b; reverse primer |
| J42 | 5'TATGATCTGCCTTCTCATCTGAAGACTAGTTTATT3' | Generation of MIR9863a(21nt) to express 21-nt shortened miR9863a; forward primer |
| J43 | 5'GATGAGAAGGCAGATCATAACAATTGAGCAACAAT3' | Generation of MIR9863a(21nt) to express 21-nt shortened miR9863a; reverse primer |
| J44 | 5'CGACGACAAGACCCTAGAACAGGATTGGGATTGA3' | Construction of pTRV2:*NbAGO1-1as*; forward primer |
| J45 | 5'GAGGAGAAGAGCCCTCATTGAGCCACTGCCGATTA3' | Construction of pTRV2:*NbAGO1-1as*; reverse primer |
| J46 | 5'CGACGACAAGACCCTGGACGCTGGCAGGTCACTTT3' | Construction of pTRV2:*NbAGO1-2as*; forward primer |
| J47 | 5'GAGGAGAAGAGCCCTCCTGTGGGTCGTTCTTTCTATT3' | Construction of pTRV2:*NbAGO1-2as*; reverse primer |
| J48 | 5'CGACGACAAGACCCTCAACAGCCCAACGCTCTAC3' | Construction of pTRV2:*NbAGO4-1as*; forward primer |
| J49 | 5'GAGGAGAAGAGCCCTGTGAAGACTGCTCCCGCTAA3' | Construction of pTRV2:*NbAGO4-1as*; reverse primer |
| J50 | 5'CGACGACAAGACCCTACATCAACGAAGTTCTTAGGGT3' | Construction of pTRV2:*NbAGO4-2as*; forward primer |
| J51 | 5'GAGGAGAAGAGCCCTATCAGAACCCGAGCCAGTG3' | Construction of pTRV2:*NbAGO4-2as*; reverse primer |
| J52 | 5'AAGGAAGTTTAAGCTATTATGATCCTATACCTTCTCAGTTGTTGTTGTTATGGTCT3' | Construction of pCaBS-γSTTM-miR9863 |
| J53 | 5'AGATCATAATTCTTCTTCTTTAGACCATATTTAAATTAGACCATAACAACAACAA3' | Construction of pCaBS-γSTTM-miR9863 |
| J54 | 5'AACCACCACCACCGTTCAGATGAGATAGAGGCAGATCATAATTCTTCTTC3' | Construction of pCaBS-γSTTM-miR9863 |
| J55 | 5' AAGGAAGTTTAAGTTGTTGTTGTTATGGTCTA 3' | Construction of pCaBS-γSTTM-EV |
| J56 | 5'AACCACCACCACCGTATTCTTCTTCTTTAGACCAT3' | Construction of pCaBS-γSTTM-EV |
| J57 | 5'CACCATGTATGCTGAAGCGACAGAG3' | Amplification of Mla1-ARC-mYFP sequence |
| J58 | 5'TGCTCACCATGGCTTGATGATCTTCATTTC3' | Amplification of Mla1-ARC-mYFP sequence |
| J59 | 5'TCATCAAGCCATGGTGAGCAAGGGCGAGGA3' | Amplification of Mla1-ARC-mYFP sequence |
| J60 | 5'CTTGTACAGCTCGTCCATGC3' | Amplification of Mla1-ARC-mYFP sequence |
| J61 | 5'GGTTCCTCGTCTCAGCCAC3' | Semi-qPCR determination of *NbAGO1* silencing; forward primer |
| J62 | 5'CTGGAAGCCTCTTCCCAAG3' | Semi-qPCR determination of *NbAGO1* silencing; reverse primer |
| J63 | 5'ATCCAGTCAAAACTTCCCG3' | Semi-qPCR determination of *NbAGO4* silencing; forward primer |
| J64 | 5'GAGATATTGAAGGCCACTGC3' | Semi-qPCR determination of *NbAGO4* silencing; reverse primer |
| J65 | 5'GGCGTGACAGGAACAAGGTATT3' | qPCR for the level of *Mla1* amplicon 1; forward primer |
| J66 | 5'CTGATTGGCATCCAGCATCG3' | qPCR for the level of *Mla1* amplicon 1; reverse primer |
| J67 | 5'TTAGAGGAGATGCGGAGAATACT3' | qPCR for the level of *Mla1* amplicon 2; forward primer |
| J68 | 5'TGCTATTCCCACTGCCATCC3' | qPCR for the level of *Mla1* amplicon 2; reverse primer |
| J69 | 5'TAACGAGCACCGACCAAACG3' | qPCR for the level of *Mla1* amplicon 3; forward primer |
| J70 | 5'ACGCCAAGGCTGAACCAAAC3' | qPCR for the level of *Mla1* amplicon 3; reverse primer |
| J71 | 5'GTCGTATCCAGTGCAGGGTCCGAGGTATTCGCACTGGATACGACgctatt3' | Reverse transcription of mature miR9863a/b.2 |
| J72 | 5'GTCGTATCCAGTGCAGGGTCCGAGGTATTCGCACTGGATACGACtatgat3' | Reverse transcription of mature miR9863c/b.1 |
| J73 | 5'CGTCGTGAGAAGGCAGATCAT3' | qPCR determination of mature miR9863a/b.2; forward primer; used with J80 |
| J74 | 5'CGGCGTCAGATGAGAAGGCAG3' | qPCR determination of mature miR9863c/b.1; forward primer; used with J80 |
| J75 | 5'GTCGTATCCAGTGCAGGGTCCGAGGTATTCGCACTGGATACGACatccga3' | Reverse transcription of phasiRNAI |
| J76 | 5'GTCGTATCCAGTGCAGGGTCCGAGGTATTCGCACTGGATACGACgcaggc3' | Reverse transcription of phasiRNAII |
| J77 | 5'GCGGCGATAGCTTGTATTTGC3' | qPCR determination of phasiRNAI; forward primer; used with J80 |
| J78 | 5'CGTCGTTGGGGAGAATAGCA3' | qPCR determination of phasiRNAII; forward primer; pad with J80 |
| J79 | 5'GTGCAGGGTCCGAGGT3' | qPCR detection of miR9863a/c/b.1/b.2 and phasiRNAI/II; reverse primer |
| J80 | 5'GGGGACATCCGATAAAATTGG3' | qPCR determination of *U6*; forward primer |
| J81 | 5'GGACCATTTCTCGATTTGTGC3' | qPCR determination of *U6*; reverse primer |
| J82 | 5'TGGCACCCGAGGAGCACC3’ | qPCR determination of *Actin*; forward primer |
| J83 | 5'GTAACCTCTCTCGGTGAG3’ | qPCR determination of *Actin*; reverse primer |
| J84 | 5'GCTATTATGATCTACCTTCTCA3' | Probe used for Northern determination of miR9863a |
| J85 | 5'TATGATCTGCCTTCTCATCTGA3' | Probe used for Northern determination of miR9863b.1 |
| J86 | 5'GCTATTATGATCTGCCTTCTCA3' | Probe used for Northern determination of miR9863b.2 |
| J87 | 5'ATATACATAGATACAGTAGACAAGTTTTCAGATGAGAAGGTA3' | Probe used for Northern determination of *Mla1* derived phasiRNAs |
| J88 | 5'GTGCTCACTCTCTTCTGTCA3' | Probe used for Northern determination of miR156a |
