# Supplementary material for: The miR9863 Family Regulates Distinct Mla Alleles in Barley to Attenuate NLR Receptor-Triggered Disease Resistance and Cell-Death Signaling
Source: PLoS Genet. 2014 Dec 11;10(12):e1004755. doi: 10.1371/journal.pgen.1004755 (PMC4263374; doi:10.1371/journal.pgen.1004755)
Supplement: S3 Table — Plasmids and constructs used in this study. (DOCX) [file pgen.1004755.s014.docx]

**Table S3. Plasmids and constructs used in this study**

| **Construct name** | **Description** | **Source/reference** |
| --- | --- | --- |
| CTAPi-GW-3HA | A *N. benthamiana* expression vector to produce 3HA fusion | [[1](#_ENREF_1)] |
| CTAPi-*Mla1*-3HA | CTAPi carrying wild type *Mla1* | This study |
| CTAPi-*Mla2*-3HA | CTAPi carrying wild type *Mla2* | This study |
| CTAPi-*Mla6*-3HA | CTAPi carrying wild type *Mla6* | This study |
| CTAPi-*Mla10-*3HA | CTAPi carrying wild type *Mla10* | This study |
| CTAPi-*Mla12*-3HA | CTAPi carrying wild type *Mla12* | This study |
| CTAPi-*Mla28*-3HA | CTAPi carrying wild type *Mla28* | This study |
| CTAPi-*Mla32*-3HA | CTAPi carrying wild type *Mla32* | This study |
| CTAPi-*Mla1*(TC1278GC)-3HA | CTAPi carrying *Mla1* with 'TC' to 'GC' mutation at 1278 nt position | This study |
| CTAPi-*Mla1*(TC1278GA)-3HA | CTAPi carrying *Mla1* with 'TC' to 'GA' mutation at 1278 nt position | This study |
| CTAPi-*Mla1*(CT1270GA)-3HA | CTAPi carrying *Mla1* with 'CT' to 'GA' mutation at 1270 nt position | This study |
| CTAPi-*Mla1*(T1266A)-3HA | CTAPi carrying *Mla1* with 'T' to 'A' mutation at 1266 nt position | This study |
| CTAPi-*Mla2*(GC1278TC)-3HA | CTAPi carrying *Mla2* with 'GC' to 'TC' mutation at 1278 nt position | This study |
| CTAPi-*Mla6*(GC1278TA)-3HA | CTAPi carrying *Mla6* with 'GC' to 'TC' mutation at 1278 nt position | This study |
| CTAPi-*Mla10*(GA1278TC)-3HA | CTAPi carrying *Mla10* with 'GA' to 'TC' mutation at 1278 nt position | This study |
| CTAPi-*Mla12*(GA1278TC)-3HA | CTAPi carrying *Mla12* with 'GA' to 'TC' mutation at 1278 nt position | This study |
| CTAPi-*Mla1ARC*-mYFP-3HA | CTAPi carrying wild type MLA1-ARC-coding sequence | This study |
| 35S-pKANNIBAL | An expression vector for miRNAs overexpression | [[2](#_ENREF_2)] |
| 35S-*tae-MIR9863a* | pKANNIBAL carrying *tae-MIR9863a* precursor | This study |
| 35S-*tae-MIR9863c* | pKANNIBAL carrying *tae-MIR9863c* precursor | This study |
| 35S-*tae-MIR9863b* | pKANNIBAL carrying *tae-MIR9863b* precursor | This study |
| 35S-*tae-MIR9863*(cluster) | pKANNIBAL carrying *tae-MIR9863* cluster sequence | This study |
| 35S-*hvu-MIR9863a* | pKANNIBAL carrying *hvu-MIR9863a* precursor | This study |
| 35S-*hvu-MIR9863b* | pKANNIBAL carrying *hvu-MIR9863b* precursor | This study |
| 35S-*aMIR9863a* | pKANNIBAL carrying artificial *MIR9863a* precursor | This study |
| 35S-*aMIR9863b.1* | pKANNIBAL carrying artificial *MIR9863b.1* precursor | This study |
| 35S-*aMIR9863b.2* | pKANNIBAL carrying artificial *MIR9863b.2* precursor | This study |
| 35S-*tae-MIR9863a*(T9C) | pKANNIBAL carrying *MIR9863a*(T9C) precursor that should produce miR9863a with a 'T' to 'C' mutation at its 9th position | This study |
| 35S-*hvu-MIR9863b*(21nt) | pKANNIBAL carrying *MIR9863b*(21nt) precursor that should produce 21-nt shortened miR9863b | This study |
| 35S-*tae-MIR9863a*(21nt) | pKANNIBAL carrying *MIR9863a*(21nt) precursor that should produce 21-nt shortened miR9863a | This study |
| pTRV1 | Plasmid for TRV mediated gene silencing | [[3](#_ENREF_3)] |
| pTRV2-LIC | An expression vector for TRV mediated gene silencing | [[4](#_ENREF_4)] |
| pTRV2-*NbPDSas* | pTRV2 carrying partial *NbPDS* anti-sense sequence | [[3](#_ENREF_3)] |
| pTRV2-*NbAGO1-1as* | pTRV2 carrying partial *NbAGO1-1* anti-sense sequence | This study |
| pTRV2-*NbAGO1-2as* | pTRV2 carrying partial *NbAGO1-2*anti-sense sequence | This study |
| pTRV2-*NbAGO4-1as* | pTRV2 carrying partial *NbAGO4-1* anti-sense sequence | This study |
| pTRV2-*NbAGO4-2as* | pTRV2 carrying partial *NbAGO4-2* anti-sense sequence | This study |
| pCaBS-α | Plasmid for BSMV mediated gene silencing | [[5](#_ENREF_5)] |
| pCaBS-β | Plasmid for BSMV mediated gene silencing | [[5](#_ENREF_5)] |
| pCaBS-γbLIC | An expression vector for BSMV mediated gene silencing | [[5](#_ENREF_5)] |
| pCaBS-γSTTM-EV | pCaBS-γbLIC carrying STTM-EV structure | This study |
| pCaBS-γSTTM-miR9863 | pCaBS-γbLIC carrying STTM-miR9863 structure | This study |
| pUbi-GATE | An expression vector for transient overexpression in barley epidermal cell | [[6](#_ENREF_6)] |
| pUbi-*tae-MIR9863a* | pUbi-GATE carrying *tae-MIR9863a* precursor | This study |
| pUbi-*tae-MIR9863c* | pUbi-GATE carrying *tae-MIR9863c* precursor | This study |
| pUbi-*hvu-MIR9863b* | pUbi-GATE carrying *hvu-MIR9863b* precursor | This study |
| pUbi-GW-mYFP | An expression vector for transient overexpression of mYFP-tagged proteins in barley epidermal cell | [[6](#_ENREF_6)] |
| pUbi-*Mla1*-mYFP | pUbi-GATE carrying mYFP-tagged wild type *Mla1* | This study |
| pUbi-*Mla10*-mYFP | pUbi-GATE carrying mYFP-tagged wild type *Mla10* | [[6](#_ENREF_6)] |
| pGY-1 | Negative control vector used in transient overexpression assay in barley epidermal cell | [[6](#_ENREF_6)] |

**References**

1. Bai S, Liu J, Chang C, Zhang L, Maekawa T, et al. (2012) Structure-function analysis of barley NLR immune receptor MLA10 reveals its cell compartment specific activity in cell death and disease resistance. PLoS Pathog 8: e1002752.

2. Li Y, Zhang Q, Zhang J, Wu L, Qi Y, et al. (2010) Identification of microRNAs involved in pathogen-associated molecular pattern-triggered plant innate immunity. Plant Physiol 152: 2222-2231.

3. Liu Y, Schiff M, Marathe R, Dinesh-Kumar SP (2002) Tobacco *Rar1*, *EDS1* and *NPR1/NIM1* like genes are required for N-mediated resistance to tobacco mosaic virus. Plant J 30: 415-429.

4. Dong Y, Burch-Smith TM, Liu Y, Mamillapalli P, Dinesh-Kumar SP (2007) A ligation-independent cloning tobacco rattle virus vector for high-throughput virus-induced gene silencing identifies roles for *NbMADS4-1* and *-2* in floral development. Plant Physiol 145: 1161-1170.

5. Yuan C, Li C, Yan L, Jackson AO, Liu Z, et al. (2011) A high throughput *Barley stripe mosaic virus* vector for virus induced gene silencing in monocots and dicots. PLoS One 6: e26468.

6. Shen QH, Saijo Y, Mauch S, Biskup C, Bieri S, et al. (2007) Nuclear activity of MLA immune receptors links isolate-specific and basal disease-resistance responses. Science 315: 1098-1103.
